# Low Divergence of *Clonorchis sinensis* in China Based on Multilocus Analysis

**DOI:** 10.1371/journal.pone.0067006

**Published:** 2013-06-18

**Authors:** Jiufeng Sun, Yan Huang, Huaiqiu Huang, Pei Liang, Xiaoyun Wang, Qiang Mao, Jingtao Men, Wenjun Chen, Chuanhuan Deng, Chenhui Zhou, Xiaoli Lv, Juanjuan Zhou, Fan Zhang, Ran Li, Yanli Tian, Huali Lei, Chi Liang, Xuchu Hu, Jin Xu, Xuerong Li, XinbingYu

**Affiliations:** 1 Guangdong Provincial Institute of Public Health, Guangdong Provincial Center for Disease Control and Prevention. Guangzhou, China; 2 Department of Parasitology, Zhongshan School of Medicine, Sun Yat-Sen University; Key Laboratory of Tropical Diseases Control, Sun Yat-sen University, Ministry of Education, Guangzhou, China; 3 Department of Dermatology and Venereology, The Third Affiliated Hospital Sun Yat-sen University, Guangzhou, China; The George Washington University Medical Center, United States of America

## Abstract

*Clonorchis sinensis*, an ancient parasite that infects a number of piscivorous mammals, attracts significant public health interest due to zoonotic exposure risks in Asia. The available studies are insufficient to reflect the prevalence, geographic distribution, and intraspecific genetic diversity of *C. sinensis* in endemic areas. Here, a multilocus analysis based on eight genes (ITS1, *act*, *tub*, *ef-1a*, *cox1*, *cox3*, *nad4* and *nad5* [4.986 kb]) was employed to explore the intra-species genetic construction of *C. sinensis* in China. Two hundred and fifty-six *C. sinensis* isolates were obtained from environmental reservoirs from 17 provinces of China. A total of 254 recognized Multilocus Types (MSTs) showed high diversity among these isolates using multilocus analysis. The comparison analysis of nuclear and mitochondrial phylogeny supports separate clusters in a nuclear dendrogram. Genetic differentiation analysis of three clusters (A, B, and C) showed low divergence within populations. Most isolates from clusters B and C are geographically limited to central China, while cluster A is extraordinarily genetically diverse. Further genetic analyses between different geographic distributions, water bodies and hosts support the low population divergence. The latter haplotype analyses were consistent with the phylogenetic and genetic differentiation results. A recombination network based on concatenated sequences showed a concentrated linkage recombination population in *cox1*, *cox3, nad4* and *nad5*, with spatial structuring in ITS1. Coupled with the history record and archaeological evidence of *C. sinensis* infection in mummified desiccated feces, these data point to an ancient origin of *C. sinensis* in China. In conclusion, we present a likely phylogenetic structure of the *C. sinensis* population in mainland China, highlighting its possible tendency for biogeographic expansion. Meanwhile, ITS1 was found to be an effective marker for tracking *C. sinensis* infection worldwide. Thus, the present study improves our understanding of the global epidemiology and evolution of *C. sinensis*.

## Introduction

*Clonorchis sinensis*, the etiological agent of clonorchiasis, is the most endemic fish-borne zoonotic liver fluke that causes a heavy socioeconomic burden in south Asia [1]. Approximately 35 million people are infected with *C. sinensis* globally, and 610 million people are at risk of infection [2]. The major endemic areas are in Asia, including China, Korea, Japan, Taiwan and Vietnam. In mainland China, an estimated 15 million people are infected with *C. sinensis* [3], [4]. The infection route and life cycle of *C. sinensis* have been determined. The first intermediate snail hosts are primarily species of *Parafossarulus* and *Bithynia*. Numerous species of freshwater fish serve as the second intermediate hosts, and piscivorous mammals, including human beings, cats and dogs, are the definitive hosts [3], [4]. Consumption of raw or inadequately cooked freshwater fish containing infective metacercariae is the routine way of contracting the disease for human beings and animal reservoirs [5].

Extensive studies of clonorchiasis over several decades in China, Korea and Japan have shown significant progress in understanding its morphological features by studying its ultra-structure, biology, pathogenesis, epidemiology and clinical manifestations [1], [3], [6]. However, the available studies have not been sufficient with respect to prevalence, geographic distribution, and the intraspecies genetic diversity of *C. sinensis*.

In an initial study, Choi [7] and Zou et al. [8] demonstrated that *C. sinensis* showed geographical differences in terms of host specificity and other biological features. Isolates from Korea utilize *Parafossarulus manchouricus* as the first intermediate host, whereas *Alocinma longicornis* serves as the first intermediate host in China isolates. Park et al. [9] demonstrated the geographical differences of *C. sinensis* from Shenyang in China and Kimhae in Korea in terms of chromosomes using karyological analysis in 2000. In the same year, isozyme electrophoresis was employed to address the different electrophoresis patterns of *C. sinensis* coming from the same region. Four of them (EST, GPD, HBDH and PGI) displayed heterozygous patterns, of which GPD was considered to be a specific genetic marker to distinguish grouped populations of *C. sinensis* from China and Korea [10]. Later, Park et al. [11] further analyzed the partial sequences of ribosomal DNA (18S and ITS2) and the mitochondrial *cox1* gene of *C. sinensis* isolated from Shenyang in China and Kimhae in Korea using multilocus sequencing. Few variations were observed from sequenced loci. Their results were confirmed by Lee et al. in 2004 using sequencing analysis of the same genes, except for an additional partial ITS1 from Kimhae in Korea and Shenyang and Guangxi in China, from which seven nucleotides of the approximate 2.6 kb sequences were found [12]. In addition, targeting the partial ribosomal DNA region of ITS1 and ITS2, Liu et al. [13] reported that only 15 nucleotide position differences were detected in the ITS1 sequence between *C. sinensis* collected from ancient and modern hosts in the same region. Based on these DNA sequences, the genetic variability among *C. sinensis* samples from different geographic origins shows negligible genetic diversity. However, using RAPD(random amplified polymorphic DNA) and MGEs(mobile genetic elements)-PCR methods, Lai et al. [14] indicated that the genetic variation of *C. sinensis* that occurred in a subtropical region (Guangdong, Guangxi and Sichuan province) developed more rapidly than that in a cold region (Heilongjiang province). However, the available studies were limited in geographical distribution. Most of the tested isolates were collected from Kimhae in Korea or in the north (Shenyang, Heilongjiang) or south (Guangdong, Guangxi, Sichuan) of China. Compared to the entire endemic region of C. sinensis, the limited scope of sampling may increase the risk of deviating from the original geographical regulation. Particularly in China, 27 of 31 provinces are endemic regions, but most of the locations were not involved in documented studies. Therefore, it would be useful to clarify the molecular epidemiology characteristics of this parasite over many geographical regions, from which we can obtain more valuable information regarding the evolution of this parasite.

The aim of this study was to explore the population genetic structure of *C. sinensis* in China, with the intention of figuring out the global patterns of *C. sinensis* infection. Our specific goals were as follows: (i) to describe the genetic structure of *C. sinensis* using multilocus analysis, (ii) to compare the population genetic structure of these isolates against the available isolates deposited to GenBank, and (iii) to look for effective genetic markers to distinguish separate populations based on genetic evidence.

## Materials and Methods

### Ethical standards

The animal experiments in this study were carried out in strict accordance with the recommendations in the Guide for the Care and Use of Laboratory Animals of the Ministry of National Institutes of Health (GB 14922.2-2011). Procedures involving vertebrate animals were reviewed and approved by Sun Yat-Sen University's Animal Care and Use Committee, and permission for sampling from vertebrates was obtained from the funding project committee and Sun Yat-Sen University's Animal Care and Use Committee.

### Parasite sampling and genomic DNA extraction

Cats and dogs were captured randomly from regions endemic for clornochiasis (15 provinces) and were then sacrificed using ether anesthesia. The liver and gallbladder were removed for *C. sinensis* adult isolation. Two hundred and twenty-four *C. sinensis*-infected animals were collected in total (Table S1). Thirty-two metacercariae were isolated from fresh water fish in Guangdong and Guangxi provinces and were developed into adults in Sprague-Dawley rats using the standard method [15]. Genomic DNA from each adult worm was extracted using a commercial DNA extraction kit (Qiagen, Germany) according to the manufacturer's instructions. Briefly, a single adult worm was suspended in a 1.5 ml microcentrifuge tube containing 200 µl of extraction buffer I. After homogenizing, proteinase K (New England Biolabs, U.K.) and RNase A (New England Biolabs, U.K.) were added to obtain final concentrations of 100 µg/ml and 20 µg/ml, respectively. After incubation for 3 h at 37°C, 200 µl Buffer II was added to this mixture, followed by incubation for 10 min at 65°C. Then, 200 µl ethanol was added to the mixture. The total mixture was moved into a spin column after vortexing. After spinning for 1 min at 8000 rpm, extra protein was removed using Buffer III, and then the DNA was washed twice with 70% ethanol, followed by centrifugation at 12000 rpm for 2 min to remove extra ethanol. DNA was recovered using 50 µl buffer EB. RNase (5 µl each, 10 mg/ml in pH 7.4 NaAC) treatment was performed at 37°C for 30 min. The DNA quantification was determined at 260 nm in a UV spectrophotometer (Shimadzu, Japan).

### DNA amplification and sequencing

Eight genes were chosen for multilocus analysis: rDNA Internal Transcribed Spacer 1 (ITS1); partial genes of actin (*act*), β-tubulin (*tub*) and elongation factor (*ef-1a*); cytochrome c oxidase subunit I (*cox1*); cytochrome c oxidase subunit III (*cox3*); NADH dehydrogenase subunit IV (*nad4*); and NADH dehydrogenase subunit V (*nad5*). PCR amplification for each gene used the primer pairs listed in Table S2. PCR was performed in a 50 µL volume of a reaction mixture containing 2.5 U Extaq (TaKaRa, Japan), 5 µL 10×PCR buffer (TaKaRa, Japan), dNTPs (10 mM), 1 µL of each primer (10 pmol) and 2 µL template DNA. Amplification was performed in an ABI PRISM 2720 thermocycler (Applied Biosystems, Foster City, U.S.A) as follows for ITS1: 95°C for 5 min, followed by 35 cycles consisting of 95°C for 45 sec, 62°C for 30 sec and 72°C for 2 min, and a delay at 72°C for 7 min. The annealing temperature was changed to 66°C, 54.8°C, 59.6°C. 55.5°C, 45.7°C, 49°C, and 49°C for *act*, *tub*, *ef-1a*, *cox1*, *cox3*, *nad4* and *nad5*, respectively. Amplicons were cleaned with GFX PCR DNA and a Gel Band Purification Kit (GE Healthcare, Buckinghamshire, U.K.). Concentrations of amplicons were estimated on the gel, photographed and analyzed by the Gel Doc XR system (Biorad), with DL2000 Ladder (Eurogentec, Seraing, Belgium) as a size and concentration marker. Amplicons were subjected to direct sequencing by PCR as follows: 95°C for 1 min, followed by 30 cycles consisting of 95°C for 10 sec, 50°C for 5 s and 60°C for 2 min. Reactions were purified with Sephadex G-50 fine (GE Healthcare Bio-Sciences AB, Uppsala, Sweden). Sequencing was performed on an ABI Prism 3730XL Sequencer using an ABI Prism BigDye™ terminator cycle sequencing kit (Applied Biosystems, Foster City, U.S.A.).

### Phylogenetic reconstruction

All of the sequences were edited using SeqMan software from the Lasergene software package (DNASTAR, Wisconsin, U.S.A.). Iterative alignment was performed automatically with manual adjustment in BioNumerics v. 4.61 (Applied Maths, Kortrijk, Belgium). A phylogenetic approach was used to investigate the relationships of 256 *C. sinensis* isolates. Phylogenetic neighbor-joining trees were inferred for nuclear and mitochondrial data separately, followed by evolution distances that were computed using the Maximum Composite Likelihood method in MEGA 4.0 [16], [17]. The percentage of replicate trees in which the associated taxa clustered together was estimated by a bootstrap test inferred from 1000 replicates. For the potential phylogenetic marker evaluation, phylogenetic UPGMA trees were inferred for each locus with the same percentage replications and bootstrap test.

### DNA polymorphic analysis and neutrality test

DNA polymorphism analysis was carried out using DnaSPv5.10.00 software [18]. A subset of *C. sinensis* strains and genotypes were used to calculate haplotype and nucleotide diversity, and Tajima's *D* neutrality test was used to distinguish between DNA sequences evolving random *versus* non-random processes [19].

### Genetic differentiation between populations

D_xy_ (the average pair-wise number of nucleotide differences per site [20]) was used to estimate divergence among population groups, while K_st_ (a weighted measure of the ratio of the average pair-wise differences within populations to the total average pair-wise differences [21]) and Snn [22], [23] (the proportion of nearest neighbors in sequence space found in the same population) were used to assess differentiation between populations. These statistics were calculated in DnaSPv5.10.00 [18], with significance levels assessed by 1000 permutations.

### Haplotype networks construction

To further explore the potential relationships between different haplotypes of the *C. sinensis*, haplotype networks were constructed for *cox1, cox3, nad4*, *nad5* and ITS1 using NETWORK4.6.1.0 (www.fluxus-engineering.com) [24] according to the manufacturer's instructions.

## Results

### Parasite isolates

**Figure 1:**
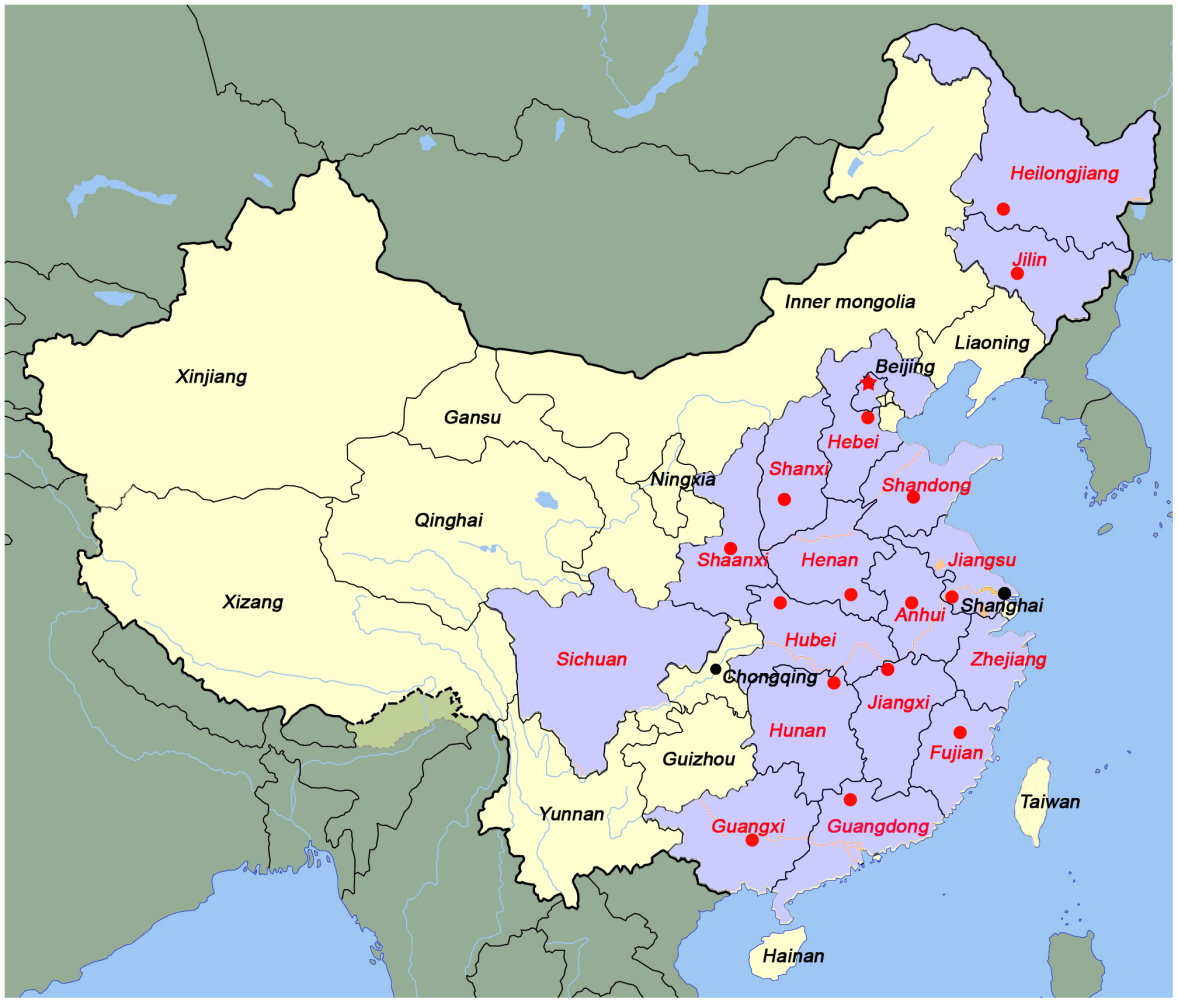
Locations where wild cats, dogs and fishes were captured. Field sites are shown in relation to the ranges of the 17 proposed provinces (marked with red and purple) in mainland China. International borders and major rivers are also shown in the figure.

The geographic distribution of the 256 isolates of *C. sinensis* is shown in Figure 1. All of the sequences that were used for multilocus calculation and phylogenetic construction were deposited into the Multilocus Sequence Typing database (http://pubmlst.org/csinensis/).

### Primer design, PCR sequencing and Multilocus analysis

The primers for ITS1 [13] and *cox1* [25] were used in previous studies, while the primers for the other genes were designed for this study. All of the primers are listed in Table S2. The primer sets proved to be specific and effective for amplifying target genes successfully in all isolates (Fig. S1). After sequencing, Blast analysis showed high homology with available sequences in the GenBank database (www.ncbi.nlm.nih.gov/genbank). The aligned sequences of the concatenated loci were 4.986 kb in total, from which 148 polymorphic sites (139 parsimony informative and 9 singleton sites) were detected (Table 1).

**Table 1:** Polymorphism summary and tests for neutral evolution in each locus.

Parameters	Phylogenetic Marker							
	ITS1	*co1*	*co3*	*nad4*	*nad5*	*ef-1a*	*act*	*tub*
No. of sequences	256	256	256	256	256	256	256	256
No. of characters	572	368	345	731	651	782	760	777
No. of cordons	n.a	80	82	208	216	258	221	243
	**DNA polymorphism analysis**							
No. of mutations (η)	12	8	14	47	32	12	16	7
Nucleotide Variability	1.22%	2.17%	4.57%	6.42%	4.91%	1.53%	2.1%	0.9%
Amino acid Variability	n.a	2.5%	3.66%	5.29%	4.62%	0	0	1.2%
No. of haplotypes	33	14	16	101	119	n.a	n.a	n.a
Haplotype diversity	0.587	0.631	0.35	0.9684	0.9707	n.a	n.a	n.a
Nucleotide diversity (Pi)	0.00128	0.00248	0.00156	0.00479	0.00531	0.00303	0.00439	0.00154
	**Neutrality test**							
Tajima's D	−1.68611 (0.05<p<0.10)	−0.64840(p>0.10)	−1.87004(p<0.05)	−1.57585(0.05<p<0.1)	−0.94367(p>0.10)	0.49353(p>0.10)	0.85054(p>0.10)	0.08993(p>0.10)

n.a: not applicable.

Seventy-six *act*, 13 *cox1*, 16 *cox3*, 29 *ef-1a*, 31 ITS1, 101 *nad4*, 119 *nad5* and 19 *tub* alleles were identified. The combination of the alleles of the eight genes resulted in a total of 254 multilocus sequence types (MSTs). MSTs 176 and 212 were shared by two isolates from Jiangsu (CSJS12, CSJS13) and Shandong province (CSSD10, CSSD15), respectively, and the other MSTs were represented by a single isolate (Table S1).

### Genetic variation and phylogeny

Parsimonious informative sites, monomorphic sites, segregating sites and the total number of mutations were calculated using DnaSPv 5.10.00 software. The results are summarized in Table 1. Nucleotide variability was higher in *cox1* (2.17%), *cox3* (4.57%), *nad4* (6.42%), and *nad5* (4.91%) than that in ITS1 (1.22%), *ef-1a* (1.53%), *act* (2.1%) and *tub* (0.9%). Amino acid variability was higher in *cox1* (2.5%), *cox3* (3.66%), *nad4* (5.29%), and *nad5* (4.62%) than that in *ef-1a* (0), *act* (0), and *tub* (1.2%).

**Figure 2:**
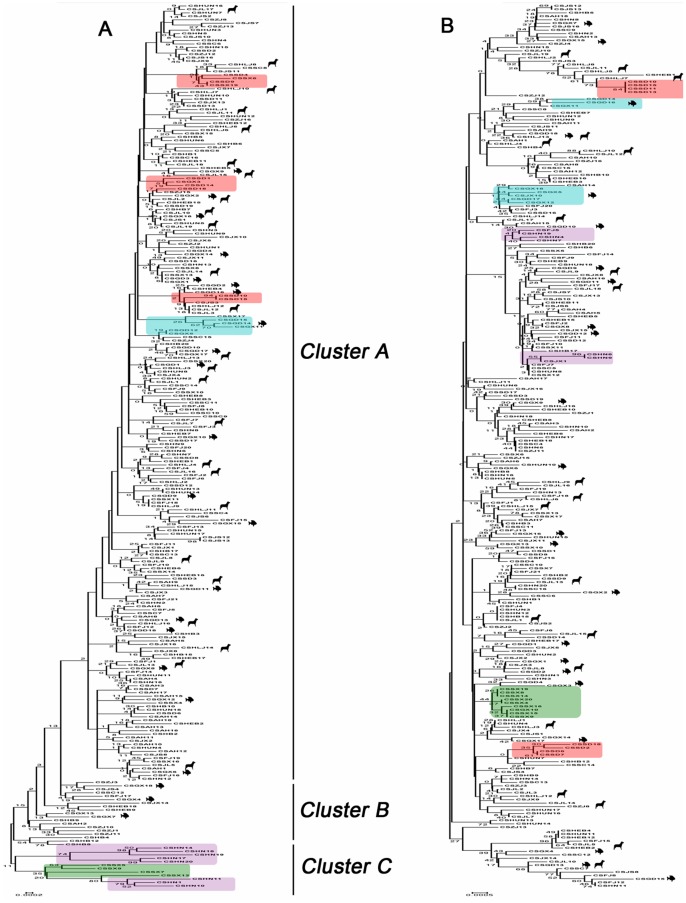
Neighbor-joining tree showing the phylogenetic relationships of the *C. sinensis* isolates included in this study (n = 256). All isolates were delineated into three major groups: the cluster A (n = 225) complex, cluster B (n = 19) and cluster C (n = 12). Geographically unique clusters were detected within the dendrogram tree: Henan (purple), Guangxi (green), Guangdong (blue) and Shandong (red). Isolates collected from fish and dogs are marked with symbols for fish and dog. The percentages of replicate trees in which the associated taxa clustered together in the bootstrap test (1000 replications) are indicated. The evolutionary distances were computed using the Maximum Composite Likelihood method and are presented in units of the number of base substitutions per site.

The relationships among the *C. sinensis* isolates were analyzed using phylogenetic algorithms of 4 nuclear and mitochondrial loci each without reference sequences. The results are shown in Figure 2, which includes two dendrograms showing the relationships among all isolates. Compared with mitochondrial DNA, the nuclear DNA-based phylogenetic tree (Fig. 2A) was delineated by three major groups within the tested isolates: the cluster A complex (n = 225), cluster B (n = 19) and cluster C (n = 12). Cluster A is composed of the most isolates from 17 provinces, and cluster B is only composed of the isolates from central China (Hubei, Anhui, Jiangsu, Hebei, Sichuan and Zhejiang) and South China (Guangxi), while cluster C was mostly found in central China (Shanxi and Henan). No structured clusters were found in mitochondrial DNA (Fig. 2B) that refer to geographic relationships. However, geographically unique groups were detected within both phylogenetic trees, such as clusters from Henan (purple), Guangxi (green), Guangdong (blue) and Shandong (red) (Fig. 2). The host-specific distribution showed no significant relationships in the mitochondrial DNA-based dendrogram (Fig. 2B). However, the isolates from cats and fish were located in cluster B in the nuclear DNA-based dendrogram (Fig. 2A), while only isolates from cats were located in cluster C.

### Disequilibrium linkage detection

Tajima's D tests the null hypothesis that populations are in mutation-drift equilibrium. In the case of significant deviation from zero, the null hypothesis of neutral (random) evolution is rejected. When this occurs, it may be due to the occurrence of natural selection or variable population dynamics. Slight deviations from neutrality were detected in the ITS1 (0.05<*p*<0.10), *cox3* (*p*<0.05) and *nad4* (0.05<*p*<0.1) loci, all of which showed negative values (Table 1).

### Genetic variability among clusters, different geographic, host species and water body distribution

The divergence and differentiation between the three separated clusters were estimated using average nucleotide divergence (Dxy) [20], a weighted measure of the ratio of the average pair-wise differences within clusters to the total average pair-wise differences (Kst) [21] and nearest-neighbor statistic (Snn) [22], [23]. Low levels of nucleotide divergence were observed, with Dxy ranging from 0.4% to 0.8%. The null hypothesis of no differentiation among sub-populations was rejected for all populations paired with each other due to significant K_st_ and S_nn_ values (Table 2). The comparison among different geographic isolates showed similar results, except that S_nn_ was not statistically significantly different between South and East China. The values of Dxy from different geographic origins of the strains were similar among South China (Guangdong, Guangxi), Northeast China (Heilongjiang, Jilin), and North China (Jiangsu, Zhejiang), while all three showed statistically significantly higher values compared with Central China (Henan, Hunan) (Table 3). The comparison among different hosts showed statistically significant differences in Kst and Snn, while there was lower support between fish and dogs in both parameters. The Dxy values of fish and dogs also exhibited the same tendency (Table 4). An additional comparison among the isolates from different bodies of water (Pearl, Yangzi, Yellow and Songhua rivers) was performed. The null hypothesis was also rejected for all populations (Table 5).

**Table 2:** Divergence and differentiation among sub-populations.

	Cluster A	Cluster B	Cluster C
No. Sequences	225	19	12
No. Segregating sites (S)	37	29	44
Average number of differences (K)	7.76278	10.18713	19.01515
Nucleotide diversity (Pi)	0.00280	0.00368	0.00686
Mutation rates(θ)	0.00281	0.00369	0.00690
	**Neutrality test**		
Tajima's D	0.46958(p>0.1)	0.90132 (p>0.1)	1.26275 (p>0.1)
Differentiation^a^^,^ b ^,^ c	Cluster A	Cluster B	Cluster C
Cluster A		0.05623***	0.09727^***^
Cluster B	0.98702^***^		0.13318^***^
Cluster C	1.00000^***^	0.98387^***^	
Cluster A			
Cluster B	0.00443		
Cluster C	0.00819	0.00681	

a K_ST_ values are displayed above the diagonal and represent the weighted measure of the ratio of the average pair-wise differences within groups to the total average pair-wise differences.

b S_nn_ values are displayed below the diagonal in bold and represent the proportion of nearest neighbors in sequence space that are found in the same group. Significance levels for K_ST_ and S_nn_ were assessed using permutation tests, with 1000 permutations: ns = non-significant, ^*^0.01<p<0.05.^**^0.001<p<0.01, ^***^p<0.001.

c Dxy values are displayed at the bottom of the diagonal and represent the minimum estimate of the number of nucleotide differences per site between groups.

**Table 3:** Divergence and differentiation among geographic distributions.

	South	North	East	Central
No. Sequences	32	33	23	38
No. Segregating sites (S)	75	82	74	104
Average number of differences (K)	16.92540	15.05303	17.47036	20.12233
Nucleotide diversity (Pi)	0.00353	0.00314	0.00364	0.00419
Mutation rates(θ)	0.00388	0.00421	0.00424	0.00516
	**Neutrality test**			
Tajima's D	−0.34186(p>0.1)	−0.95434(p>0.1)	−0.55673(p>0.1)	−0.69051(p>0.1)
Differentiation^a^^,^ b ^,^ c	South	North	East	Central
South		0.01862^**^	0.0270^*^	0.02528 ^***^
North	0.61718^*^		0.02441^***^	0.03626 ^***^
East	**0.60606 ** ns	0.63263^*^		0.01375^*^
Central	0.77119 ^***^	0.73065^***^	0.76230 ^***^	
South				
North	0.00346			
East	0.00366	0.00356		
Central	0.00406	0.00394	0.00403	

a K_ST_ values are displayed above the diagonal and represent the weighted measure of the ratio of the average pair-wise differences within groups to the total average pair-wise differences.

b S_nn_ values are displayed below the diagonal in bold and represent the proportion of nearest neighbors in sequence space that are found in the same group. Significance levels for K_ST_ and S_nn_ were assessed using permutation tests, with 1000 permutations:

ns = non-significant, ^*^0.01<p<0.05.^**^0.001<p<0.01, ^***^p<0.001.

c Dxy values are displayed at the bottom of the diagonal and represent the minimum estimate of the number of nucleotide differences per site between groups.

**Table 4 pone-0067006-t004:** Divergence and differentiation among different hosts.

	Cat	Fish	Dog
No. Sequences	191	32	33
No. Segregating sites (S)	157	75	82
Average number of differences (K)	18.57085	16.92540	15.05303
Nucleotide diversity (Pi)	0.00387	0.00353	0.00314
Mutation rates(θ)	0.00388	0.00354	0.00314
	**Neutrality test**		
Tajima's D	−0.34186(p>0.1)	−0.95434(p>0.1)	−1.03550 (p>0.1)
Differentiation^a^^,^ b ^,^ c	Cat	Fish	Dog
Cat		0.00556^***^	0.00721^***^
Fish	**0.82586** ^**^		0.01862^**^
Dog	**0.79594** ^*^	**0.61718** ^*^	
Cat			
Fish	0.00379		
Dog	0.00361	0.00346	

a K_ST_ values are displayed above the diagonal and represent the weighted measure of the ratio of the average pair-wise differences within groups to the total average pair-wise differences.

b S_nn_ values are displayed below the diagonal in bold and represent the proportion of nearest neighbors in sequence space that are found in the same group. Significance levels for K_ST_ and S_nn_ were assessed using permutation tests, with 1000 permutations: ns = non-significant, ^*^0.01<p<0.05.^**^0.001<p<0.01, ^***^p<0.001.

c Dxy values are displayed at the bottom of the diagonal and represent the minimum estimate of the number of nucleotide differences per site between groups.

**Table 5:** Divergence and differentiation among water bodies.

	Songhua River	Yellow River	Yangzi River	Pearl River
No. Sequences	33	70	87	32
No. Segregating sites (S)	82	130	117	75
Average number of differences (K)	15.05303	21.17723	16.55654	16.92540
Nucleotide diversity (Pi)	0.00314	0.00441	0.00345	0.00353
Mutation rates(θ)	0.00421	0.00571	0.00488	0.00388
	**Neutrality test**			
Tajima's D	−0.95434(p>0.1)	−0.78167 (p>0.1)	−0.98406 (p>0.1)	−0.34186 (p>0.1)
Differentiation^a^^,^ b ^,^ c	Songhua River	Yellow River	Yangzi River	Pearl River
Songhua River		0.01383^***^	0.01708^***^	0.01862^***^
Yellow River	0.71123^**^		0.00973^***^	0.01366^**^
Yangzi River	0.69639^**^	0.70456^***^		0.01243^**^
Pearl River	0.61718^*^	0.79330^**^	0.69860^*^	
Songhua River				
Yellow River	0.00390			
Yangzi River	0.00344	0.00401		
Pearl River	0.00346	0.00410	0.00360	

a K_ST_ values are displayed above the diagonal and represent the weighted measure of the ratio of the average pair-wise differences within groups to the total average pair-wise differences.

b S_nn_ values are displayed below the diagonal in bold and represent the proportion of nearest neighbors in sequence space that are found in the same group. Significance levels for K_ST_ and S_nn_ were assessed using permutation tests, with 1000 permutations: ns = non-significant, ^*^0.01<p<0.05.^**^0.001<p<0.01, ^***^p<0.001.

c Dxy values are displayed at the bottom of the diagonal and represent the minimum estimate of the number of nucleotide differences per site between groups.

### Screening reliable genetic markers to distinguish separate populations

**Figure 3:**
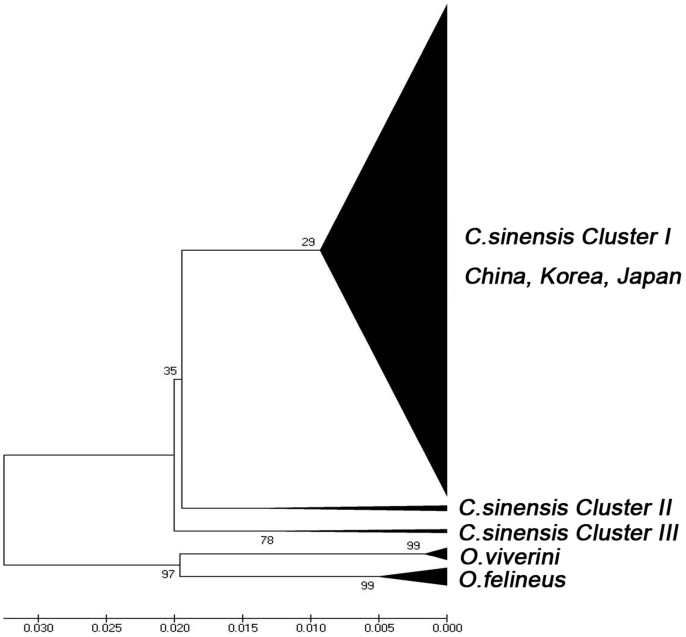
Phylogram constructed from UPGMA analysis of p-distances based on concatenated sequences of the ITS1 locus using global penal isolates (n = 288, *C. sinensis*: 271, *O. viverrini*: 7, *O. felineus*: 10). The fixed cluster I (red) is composed of 234 isolates from 17 provinces in China and 14 isolates from Korea and Japan. Cluster II (light blue) is composed of 16 isolates from Henan, Shannxi, Zhejiang, Guangxi, Anhui and Hubei province. Cluster III (light red) is composed of 6 isolates from Henan province and an ancient corpse from Hubei province in 176 BC. Seventeen *O. viverrini* (purple) and *O. felineus* (green) were used as outgroups. The percentage replicates are 1000 replications. The evolutionary distances were computed using the Maximum Composite Likelihood method.

Eight separate phylogenetic trees were constructed for the 256 *C. sinensis* isolates to evaluate genetic markers that may be used to distinguish separate clusters. The reference sequences of the *cox1* and ITS1 loci in Korea, Japan and from one ancient corpse in China were taken from the GenBank database. ITS1 sequences of *O. viverrini* and *O. felineus* were used as outgroups in the ITS1 phylogenetic tree. Three subclades corresponding to clusters I, II and III showed bootstrap support in the ITS1 locus (bootstrap value >70) (Fig. 3), while no fixed clusters were observed in other loci (data not shown). In the phylogenetic tree of ITS1, cluster I was composed of 234 isolates from 16 provinces in China and 14 isolates from Korea. Cluster II was composed of 16 isolates from the Henan, Shannxi, Zhejiang, Guangxi, Anhui and Hubei provinces. Cluster III was composed of 6 isolates from Henan province and an ancient corpse from Hubei province in 176 BC. These data were consistent with the phylogenetic results of combined nuclear genes.

### Haplotype networks analysis of *C. sinensis*

**Figure 4:**
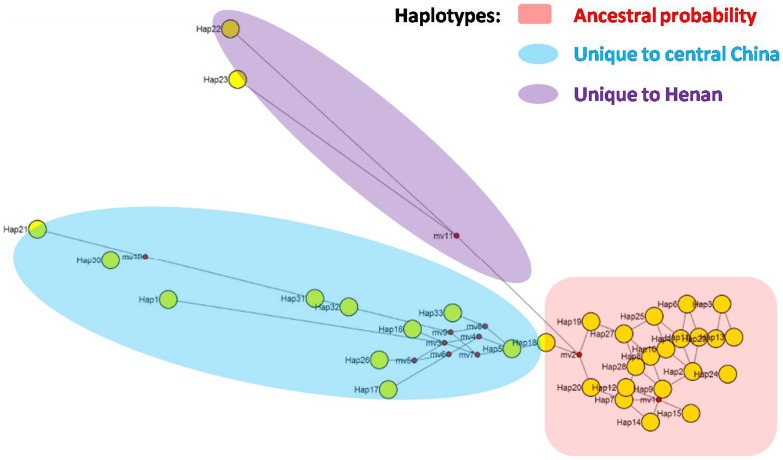
Haplotype networks of 288 isolates at ITS1 locus. The sampled haplotypes are indicated by circles; the geographical regions from which the sample was collected and the size of the circles are proportional to the observed haplotype frequency. The super linkage populations depict the haplotype with the highest ancestral probability (light red), and each branch indicates mutational separation (light blue and purple). Internal nodes (yellow) are representative of ancestral haplotypes. Media Vector (mv, red) indicate the vector between two ancestral haplotypes.

To further explore the potential relationships of *C. sinensis* in China, haplotype networks were constructed for *cox1, cox3, nad4*, *nad5* (Fig. S2) and ITS1 (Fig. 4). Sampled haplotypes are indicated by circles. According to the instructions of NETWORK4.6.1.0, the super linkage populations depict the haplotype with the highest ancestral probability, while each branch indicates mutational separation. Internal nodes (yellow) are representative of ancestral haplotypes. Media Vector (mv, red) indicates the probable vector between two haplotypes. A super linkage population was observed for the *cox1, cox3, nad4* and *nad5* loci, which represented a lower population expanding tendency (Fig. S2), while for the ITS1 locus (Fig. 4), two branches were separated from a super linkage population (marked with red), corresponding to clusters II (samples from central and south of China) and III (samples from central of China) in the ITS1 phylogenetic tree (Fig. 3). A separated branch (marked with blue) comprised the same samples from cluster II expect for CsHB9, while another branch (marked with purple) comprised the same samples from cluster III. Additionally, the samples collected from central China were positioned throughout the networks. These results indicated that the genetic expansion of the population of *C. sinensis* most likely originated from central China. The host-specific haplotype detection showed that two separate branches (hap22, hap23 and hap1, hap5, hap16–18, hap21, hap26, and hap30–33) only include the isolates collected from cats, while the major haplotype cluster contains the isolates collected from fish, cats and dogs (Fig. 4).

## Discussion

Human clonorchiasis has been reported in most provinces of China, except inner Mongolia, Ningxia, Qinghai, Tibet, and Xinjiang [3]. In Guangdong province, which is located in southern China, clonorchiasis was detected in 62 of 95 counties or cities by epidemiological surveys from 1973 to 2003. Furthermore, an analysis of coprological examination results showed that the percentage of infected people was as high as 18% [3]. The high rates of infection, ineffective therapy and relapses are included in a combination of factors that lead to the liver damage that is associated with heavy, chronic infection, which in some cases can be fatal [3]. Epidemiological typing is the key to elucidating the population structure of this pathogen in order to understand the contribution of the pathogen's genotype to the epidemiology. Therefore, the present study offers the potential to facilitate efforts to increase our knowledge and surveillance of this pathogenic parasite.

Multilocus analysis was initially used to describe the genetic structure of *C. sinensis* in mainland China. Eight loci belonging to nuclear, ribosome or mitochondrial DNA were employed for multilocus analysis. The sequences of nuclear ribosomal DNA and mitochondrial DNA have been widely used to analyze the genetic variations of *C. sinensis* and several closely relelated trematodes and cestodes [26], [27], such as *Paragonimus* [28], [29], *Schistosoma japonicum* [25], and *Echinostoma* [30]. The multilocus data showed that 76 *act*, 13 *cox1*, 16 *cox3*, 29 *ef-1a*, 31 ITS1, 101 *nad4*, 119 *nad5*, and 19 *tub* alleles were identified from 8 loci. A low diversity of alleles was detected for *act*, *ef-1a*, *tub* and ITS1 loci, while it was not found in mitochondrial genes. Much higher diversity was detected in *nad4* and *nad5* loci but not in *cox1* or *cox3* loci. It seems that the latter is more conserved in *C. sinensis*. However, Liu et al. [31] reported that *nad1* and *nad2* exhibited lower diversity than *cox1* and *cox3* in the isolates from Guangdong. The geographic difference might account for this minor difference. The results of Tajima's D test showed that ITS1, *cox3* and *nad4* were slightly non-neutral compared with the other loci, and the possibility of neutrality was not rejected within any of the geographically defined population groups, according to the latter Tajima's D values based on different clusters (Table 2).

The phylogenetic analysis showed lower divergence of all tested isolates. However, geographic variation was detected between three clusters. Cluster A consists of the majority of isolates from all endemic regions, including Guangdong and Heilongjiang provinces (3000 km apart). Clusters B and C were isolates that were primarily isolated in central China. Previous studies [21]–[28] focusing on geographic comparisons showed low divergence among isolates from Guangdong, Heilongjiang and Korea. Because the isolates from these studies are mostly located in cluster A in our study, it is therefore not surprising that all of the results of these studies showed few or no differences between tested isolates. The limited geographic isolates in these studies perhaps missed the intraspecies phylogenetic structure.

Our analysis then focused on comparing the type and distribution of diversity between the different clusters, geographic distribution, host species and water bodies of *C. sinensis*. Estimates of the average number of differences (K), nucleotide diversity (Pi) and mutation rates (θ) were consistently greater for cluster C than for the others. The genetic differences in all *C. sinensis* clusters detected in the analysis of pair-wise population combinations showed that the different clusters of *C. sinensis* were experiencing divergent evolutionary trajectories. The population genetic differences according to geographic distribution, water bodies or host species support the evolutionary divergence among the tested isolates (Tables 3–5). Therefore, high diversity, low population differences based on water bodies or host species, and geographically defined structures in tested isolations were all consistent with a model of slow population expansion. The genetic relationships between the subdivided clusters led us to examine the potential relationships of *C. sinensis* using haplotype network analysis.

Compared with a lower expanding present as a super linkage population in cox1, cox3, nad4 and nad5 loci, the separated branches in ITS1 indicated genetic separation. Two branches in the ITS1 locus corresponded to the two separate clusters in the ITS1 dendrogram. A multilocus network showed that haplotypes unique to clusters II and III were occupied by both internal and apical positions within the networks. These data are persuasive evidence for the derivation of these lineages and likely point to an origin population from which other haplotypes derived (Fig. 4). Regarding the geographic distribution of samples in each cluster in both the ITS1 dendrogram and haplotype networks, the data indicated that the genetic expansion of the *C. sinensis* population most likely originates in central China. Historical records and archaeological evidence support this speculation. *C. sinensis* was first discovered by a Chinese carpenter in India in 1875 [32], while the first autochthonous case reported in China was in 1908 [33]. Most cases of *C. sinensis* infection occurring in non-endemic countries are caused by the immigration of humans or fish trading, indicating that human activities are a vector that contributes to the expansion of the endemic region of *C. sinensis* [34], [35], [36]. Archaeological evidence in desiccated feces found in mummies mirrors these findings. From 1956 to 1994 AD, archaeological studies found *C. sinensis* eggs in desiccated feces from mummies linked to historical periods from the Warring States era (475 BC) to the Ming Dynasty (AD 1558) (Table 6) [37], [38], [39], which indicates that human clonorchiasis was present in China at least 2300 years ago. The burial time of the mummies was concurrent with the territorial expansion of each dynasty (Table 6). For instance, the territory in the Warring States period (475-221 BC) was limited to central China (Hubei, Henan, Shanxi, and Hebei provinces), while, the territory from the Han to the Song Dynasties expanded from central to south China (Fujian and Guangdong provinces). Therefore, the expansion of clonorchiasis most likely followed the migration of humans in each dynasty. Another interesting finding from the haplotype analysis is that host-specific haplotypes were detected in two separate branches (hap22, hap23 and hap1, hap5, hap16–18, hap21, hap26, hap30–33) that only included the isolates collected from cats. However, we still could not confirm whether there are internal host-parasite associations within these haplotypes or other factors related to geography. Further animal infection experiments are perhaps needed to address this issue.

**Table 6:** Summarized archaeological evidence of *C. sinensis* eggs in desiccated fecal remains in mummies according to archived archaeology studies.

Number	Gender	Age	Origin of isolation	Time of isolation	Buried time
1	Female	80	Guangzhou, Guangdong	1956 AD	Ming dynasty, 1505 AD
2	Male	50	Hengyang, Hunan	1973 AD	Song dynasty, 960–1127 AD
3	Male	60	Jiangling, Hubei	1975 AD	Han dynasty, 167 BC
4	Male	50	Fuqing, Fujian	1980 AD	Ming dynasty, 1558 AD
5	Female	50–60	Fuzhou, Fujian	1980 AD	Ming dynasty, ∼1558 AD
6	Female	40–45	Jiangling, Hubei	1982 AD	The Warring States, 475–221 BC
7	Female	70–75	Jingmen, Hubei	1994 AD	The Warring States, 475–221 BC

In addition, the constructed haplotype network of ITS1 provided evidence that it could serve as a genetic marker to distinguish separate clusters. This result is consistent with Tatonova et al.'s study [40] that demonstrated the feasibility of complete ITS1 sequences used for *C. sinensis* population genetics. The constructed ITS1 phylogenetic tree that involved most of the isolates from Korea, Japan and one ancient corpse demonstrates that it could reflect the trends in the multilocus pattern, while the *cox1* gene, which is normally used as a marker gene in animals [41], showed insufficient intraspecific variation in *C. sinensis*.

In summary, the present study delineated the geographically associated intraspecies phylogeny structure within isolates of *C. sinensis* from mainland China and highlights the possibility of this agent undergoing biogeographic expansion from central to Southern and Northern China. Thus, the drafting of multiple preventative strategies is necessary for the surveillance and prevention of *C. sinensis* infection. Meanwhile, we proved that ITS1 could be used an effective marker to track the expansion of each population of *C. sinensis* globally. Further collaborative community efforts to integrate such multilocus sequence typing approaches will lead to a better understanding of the evolution of this increasingly important, understudied, emerging human pathogenic parasite. In particular, coupled with the available genome data of C. sinensis [42], such attempts will facilitate our understanding of the global epidemiology of clonorchiasis.

## Supporting Information

Figure S1 **Electrophoresis analysis of partial results of PCR amplification in twelve genes using the primer sets in this study.**
(TIF)

Figure S2 **The haplotype networks constructed for** ***cox1, cox3, nad4*** **, and** ***nad5*** **.**
(TIF)

Table S1 **The MST profiles of the 256** ***Clonorchis sinensis*** **isolates from 17 provinces of China typed in this study.**
(DOC)

Table S2 **Primers used in this study.**
(DOC)
