# Supplementary figures and images for: Low Divergence of Clonorchis sinensis in China Based on Multilocus Analysis
Source: PLoS One. 2013 Jun 18;8(6):e67006. doi: 10.1371/journal.pone.0067006 (PMC3688995; doi:10.1371/journal.pone.0067006)

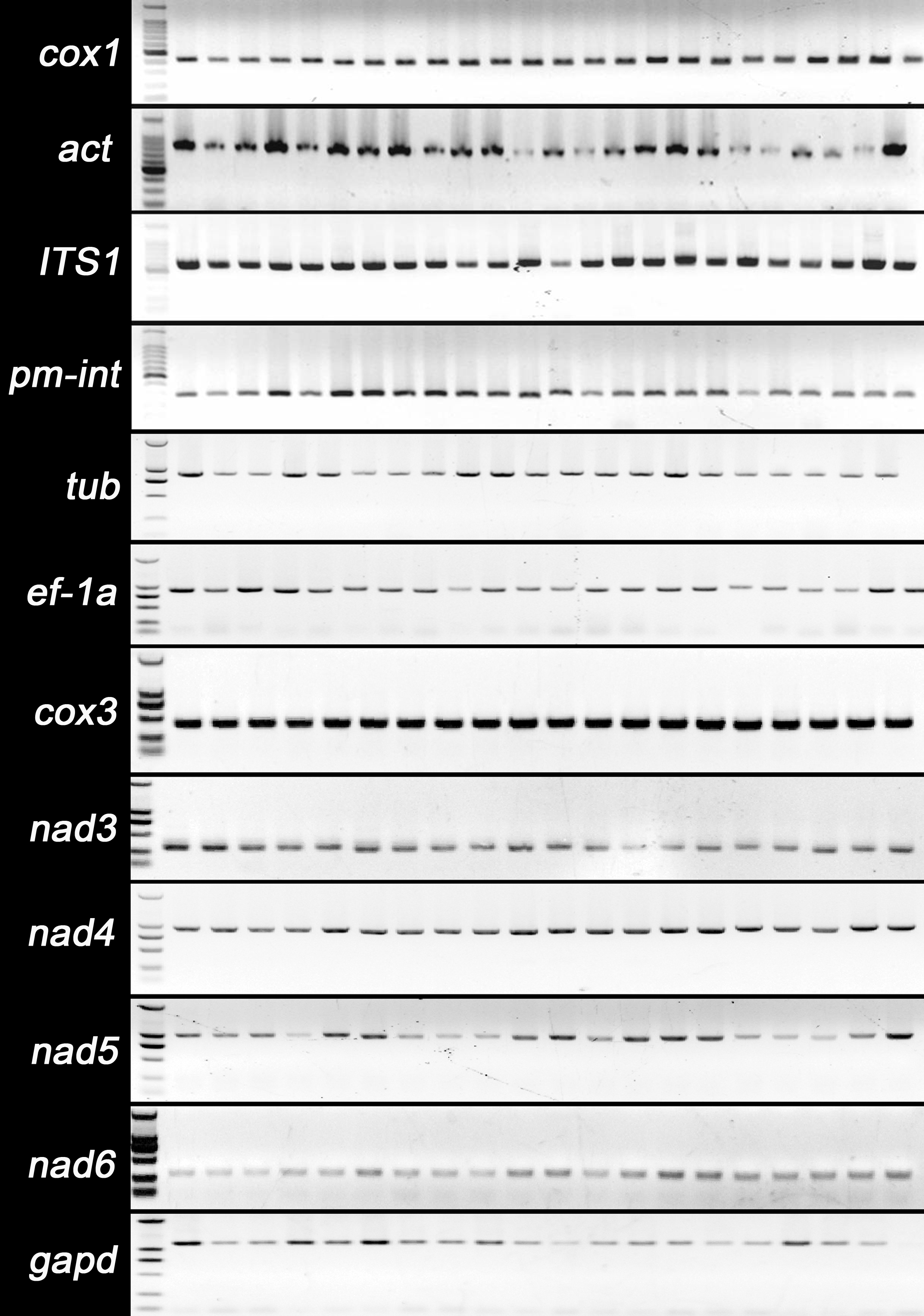

Supplement: Figure S1 — Electrophoresis analysis of partial results of PCR amplification in twelve genes using the primer sets in this study. (TIF) [file pone.0067006.s001.tif]

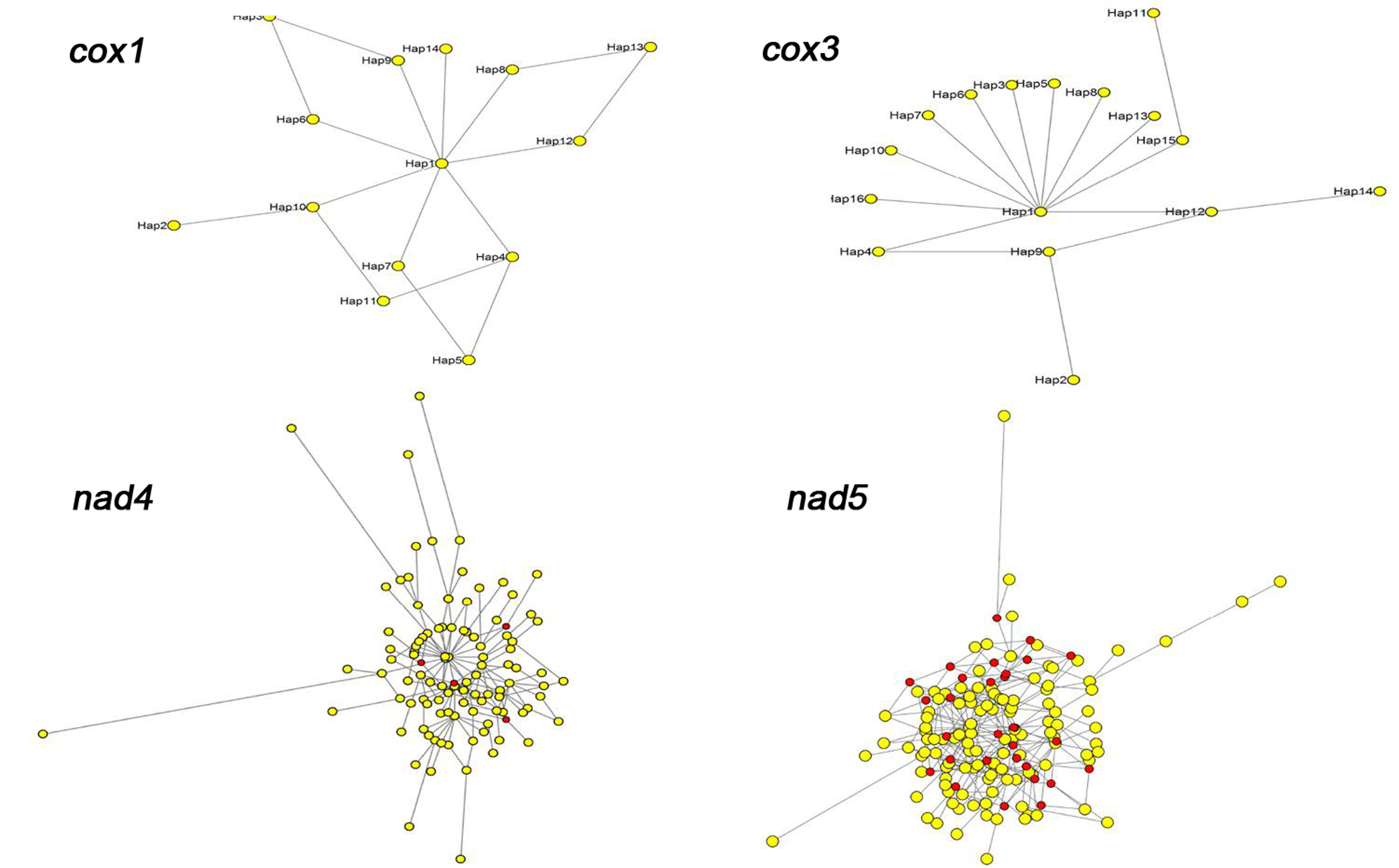

Supplement: Figure S2 — The haplotype networks constructed for cox1, cox3, nad4 , and nad5 . (TIF) [file pone.0067006.s002.tif]
