# Supplementary material for: Low Divergence of Clonorchis sinensis in China Based on Multilocus Analysis
Source: PLoS One. 2013 Jun 18;8(6):e67006. doi: 10.1371/journal.pone.0067006 (PMC3688995; doi:10.1371/journal.pone.0067006)
Supplement: Table S1 — The MST profiles of the 256 Clonorchis sinensis isolates from 17 provinces of China typed in this study. (DOC) [file pone.0067006.s003.doc]

Table S1. The MST profiles of the 256 *Clonorchis sinensis* isolates from 17 provinces of China typed in this study.

| **Isolate fields** | | | **MLST** | | | | | | | | |
| --- | --- | --- | --- | --- | --- | --- | --- | --- | --- | --- | --- |
| **id** | **isolate** | **Isolate origin** | ***act*** | ***cox1*** | ***cox3*** | ***ef-1a*** | **ITS1** | ***nad4*** | ***nad5*** | ***tub*** | **ST** |
| [1](http://pubmlst.org/perl/private/bigsdb/bigscurate.pl?page=info&db=pubmlst_csinensis_isolates&id=1) | CSAH1 | Cat, Hefei, Anhui, China | 1 | 1 | 1 | 1 | 1 | 1 | 1 | 1 | 1 |
| [2](http://pubmlst.org/perl/private/bigsdb/bigscurate.pl?page=info&db=pubmlst_csinensis_isolates&id=2) | CSAH10 | Cat, Hefei, Anhui, China | 2 | 1 | 1 | 2 | 1 | 2 | 2 | 1 | 2 |
| [3](http://pubmlst.org/perl/private/bigsdb/bigscurate.pl?page=info&db=pubmlst_csinensis_isolates&id=3) | CSAH11 | Cat, Hefei, Anhui, China | 2 | 1 | 1 | 3 | 1 | 3 | 3 | 1 | 3 |
| [4](http://pubmlst.org/perl/private/bigsdb/bigscurate.pl?page=info&db=pubmlst_csinensis_isolates&id=4) | CSAH12 | Cat, Hefei, Anhui, China | 3 | 1 | 1 | 2 | 2 | 2 | 4 | 1 | 4 |
| [5](http://pubmlst.org/perl/private/bigsdb/bigscurate.pl?page=info&db=pubmlst_csinensis_isolates&id=5) | CSAH13 | Cat, Hefei, Anhui, China | 3 | 2 | 1 | 3 | 1 | 4 | 5 | 3 | 5 |
| [6](http://pubmlst.org/perl/private/bigsdb/bigscurate.pl?page=info&db=pubmlst_csinensis_isolates&id=6) | CSAH14 | Cat, Hefei, Anhui, China | 4 | 3 | 1 | 2 | 1 | 5 | 6 | 4 | 6 |
| [7](http://pubmlst.org/perl/private/bigsdb/bigscurate.pl?page=info&db=pubmlst_csinensis_isolates&id=7) | CSAH15 | Cat, Hefei, Anhui, China | 5 | 1 | 1 | 4 | 1 | 6 | 7 | 4 | 7 |
| [8](http://pubmlst.org/perl/private/bigsdb/bigscurate.pl?page=info&db=pubmlst_csinensis_isolates&id=8) | CSAH16 | Cat, Hefei, Anhui, China | 1 | 4 | 1 | 2 | 1 | 7 | 8 | 5 | 8 |
| [9](http://pubmlst.org/perl/private/bigsdb/bigscurate.pl?page=info&db=pubmlst_csinensis_isolates&id=9) | CSAH17 | Cat, Hefei, Anhui, China | 5 | 1 | 1 | 3 | 3 | 8 | 9 | 1 | 9 |
| [10](http://pubmlst.org/perl/private/bigsdb/bigscurate.pl?page=info&db=pubmlst_csinensis_isolates&id=10) | CSAH18 | Cat, Hefei, Anhui, China | 2 | 2 | 1 | 3 | 1 | 4 | 10 | 5 | 10 |
| [11](http://pubmlst.org/perl/private/bigsdb/bigscurate.pl?page=info&db=pubmlst_csinensis_isolates&id=11) | CSAH2 | Cat, Hefei, Anhui, China | 6 | 1 | 1 | 1 | 4 | 9 | 11 | 1 | 11 |
| [12](http://pubmlst.org/perl/private/bigsdb/bigscurate.pl?page=info&db=pubmlst_csinensis_isolates&id=12) | CSAH3 | Cat, Hefei, Anhui, China | 5 | 1 | 1 | 3 | 1 | 10 | 12 | 2 | 12 |
| [13](http://pubmlst.org/perl/private/bigsdb/bigscurate.pl?page=info&db=pubmlst_csinensis_isolates&id=13) | CSAH4 | Cat, Hefei, Anhui, China | 5 | 4 | 1 | 5 | 1 | 11 | 13 | 1 | 13 |
| [14](http://pubmlst.org/perl/private/bigsdb/bigscurate.pl?page=info&db=pubmlst_csinensis_isolates&id=14) | CSAH5 | Cat, Hefei, Anhui, China | 7 | 5 | 1 | 5 | 2 | 11 | 13 | 6 | 14 |
| [15](http://pubmlst.org/perl/private/bigsdb/bigscurate.pl?page=info&db=pubmlst_csinensis_isolates&id=15) | CSAH6 | Cat, Hefei, Anhui, China | 8 | 1 | 1 | 5 | 1 | 6 | 14 | 7 | 15 |
| [16](http://pubmlst.org/perl/private/bigsdb/bigscurate.pl?page=info&db=pubmlst_csinensis_isolates&id=16) | CSAH7 | Cat, Hefei, Anhui, China | 9 | 6 | 1 | 3 | 1 | 12 | 15 | 6 | 16 |
| [17](http://pubmlst.org/perl/private/bigsdb/bigscurate.pl?page=info&db=pubmlst_csinensis_isolates&id=17) | CSAH8 | Cat, Hefei, Anhui, China | 10 | 1 | 1 | 5 | 1 | 13 | 16 | 2 | 17 |
| [18](http://pubmlst.org/perl/private/bigsdb/bigscurate.pl?page=info&db=pubmlst_csinensis_isolates&id=18) | CSAH9 | Cat, Hefei, Anhui, China | 8 | 7 | 1 | 5 | 1 | 17 | 14 | 8 | 18 |
| [19](http://pubmlst.org/perl/private/bigsdb/bigscurate.pl?page=info&db=pubmlst_csinensis_isolates&id=19) | CSFJ1 | Cat, Nanping, Fujian, China | 11 | 6 | 1 | 5 | 1 | 4 | 14 | 1 | 19 |
| [20](http://pubmlst.org/perl/private/bigsdb/bigscurate.pl?page=info&db=pubmlst_csinensis_isolates&id=20) | CSFJ10 | Cat, Nanping, Fujian, China | 2 | 4 | 1 | 6 | 1 | 15 | 18 | 1 | 20 |
| [21](http://pubmlst.org/perl/private/bigsdb/bigscurate.pl?page=info&db=pubmlst_csinensis_isolates&id=21) | CSFJ11 | Cat, Nanping, Fujian, China | 12 | 4 | 1 | 2 | 1 | 16 | 19 | 1 | 21 |
| [22](http://pubmlst.org/perl/private/bigsdb/bigscurate.pl?page=info&db=pubmlst_csinensis_isolates&id=22) | CSFJ12 | Cat, Nanping, Fujian, China | 6 | 1 | 2 | 5 | 1 | 17 | 20 | 2 | 22 |
| [23](http://pubmlst.org/perl/private/bigsdb/bigscurate.pl?page=info&db=pubmlst_csinensis_isolates&id=23) | CSFJ13 | Cat, Nanping, Fujian, China | 13 | 1 | 1 | 7 | 1 | 18 | 21 | 4 | 23 |
| [24](http://pubmlst.org/perl/private/bigsdb/bigscurate.pl?page=info&db=pubmlst_csinensis_isolates&id=24) | CSFJ14 | Cat, Nanping, Fujian, China | 14 | 4 | 3 | 5 | 1 | 19 | 22 | 1 | 24 |
| [25](http://pubmlst.org/perl/private/bigsdb/bigscurate.pl?page=info&db=pubmlst_csinensis_isolates&id=25) | CSFJ15 | Cat, Nanping, Fujian, China | 15 | 8 | 4 | 8 | 5 | 20 | 23 | 6 | 25 |
| [26](http://pubmlst.org/perl/private/bigsdb/bigscurate.pl?page=info&db=pubmlst_csinensis_isolates&id=26) | CSFJ16 | Cat, Nanping, Fujian, China | 13 | 1 | 1 | 5 | 1 | 21 | 14 | 1 | 26 |
| [27](http://pubmlst.org/perl/private/bigsdb/bigscurate.pl?page=info&db=pubmlst_csinensis_isolates&id=27) | CSFJ17 | Cat, Nanping, Fujian, China | 2 | 4 | 5 | 5 | 6 | 22 | 24 | 4 | 27 |
| [28](http://pubmlst.org/perl/private/bigsdb/bigscurate.pl?page=info&db=pubmlst_csinensis_isolates&id=28) | CSFJ18 | Cat, Nanping, Fujian, China | 3 | 6 | 1 | 5 | 1 | 23 | 25 | 1 | 28 |
| [29](http://pubmlst.org/perl/private/bigsdb/bigscurate.pl?page=info&db=pubmlst_csinensis_isolates&id=29) | CSFJ19 | Cat, Nanping, Fujian, China | 16 | 6 | 1 | 3 | 1 | 24 | 14 | 1 | 29 |
| [30](http://pubmlst.org/perl/private/bigsdb/bigscurate.pl?page=info&db=pubmlst_csinensis_isolates&id=30) | CSFJ2 | Cat, Nanping, Fujian, China | 17 | 4 | 1 | 9 | 1 | 15 | 26 | 6 | 30 |
| [31](http://pubmlst.org/perl/private/bigsdb/bigscurate.pl?page=info&db=pubmlst_csinensis_isolates&id=31) | CSFJ20 | Cat, Nanping, Fujian, China | 2 | 1 | 6 | 10 | 1 | 6 | 11 | 6 | 31 |
| [32](http://pubmlst.org/perl/private/bigsdb/bigscurate.pl?page=info&db=pubmlst_csinensis_isolates&id=32) | CSFJ21 | Cat, Nanping, Fujian, China | 18 | 1 | 1 | 3 | 1 | 6 | 27 | 6 | 32 |
| [33](http://pubmlst.org/perl/private/bigsdb/bigscurate.pl?page=info&db=pubmlst_csinensis_isolates&id=33) | CSFJ3 | Cat, Nanping, Fujian, China | 19 | 1 | 6 | 11 | 7 | 6 | 1 | 1 | 33 |
| [34](http://pubmlst.org/perl/private/bigsdb/bigscurate.pl?page=info&db=pubmlst_csinensis_isolates&id=34) | CSFJ4 | Cat, Nanping, Fujian, China | 8 | 1 | 1 | 12 | 1 | 6 | 1 | 1 | 34 |
| [35](http://pubmlst.org/perl/private/bigsdb/bigscurate.pl?page=info&db=pubmlst_csinensis_isolates&id=35) | CSFJ5 | Cat, Nanping, Fujian, China | 20 | 4 | 7 | 5 | 1 | 25 | 28 | 7 | 35 |
| [36](http://pubmlst.org/perl/private/bigsdb/bigscurate.pl?page=info&db=pubmlst_csinensis_isolates&id=36) | CSFJ6 | Cat, Nanping, Fujian, China | 18 | 1 | 1 | 13 | 1 | 26 | 1 | 1 | 36 |
| [37](http://pubmlst.org/perl/private/bigsdb/bigscurate.pl?page=info&db=pubmlst_csinensis_isolates&id=37) | CSFJ7 | Cat, Nanping, Fujian, China | 21 | 4 | 1 | 10 | 1 | 15 | 28 | 1 | 37 |
| [38](http://pubmlst.org/perl/private/bigsdb/bigscurate.pl?page=info&db=pubmlst_csinensis_isolates&id=38) | CSFJ8 | Cat, Nanping, Fujian, China | 22 | 1 | 2 | 1 | 1 | 17 | 29 | 1 | 38 |
| [39](http://pubmlst.org/perl/private/bigsdb/bigscurate.pl?page=info&db=pubmlst_csinensis_isolates&id=39) | CSFJ9 | Cat, Nanping, Fujian, China | 23 | 4 | 1 | 5 | 3 | 19 | 30 | 1 | 39 |
| [40](http://pubmlst.org/perl/private/bigsdb/bigscurate.pl?page=info&db=pubmlst_csinensis_isolates&id=40) | CSGD1 | Fish, Yangshan, Guangdong,China | 8 | 1 | 1 | 2 | 1 | 4 | 31 | 6 | 40 |
| [41](http://pubmlst.org/perl/private/bigsdb/bigscurate.pl?page=info&db=pubmlst_csinensis_isolates&id=41) | CSGD10 | Fish, Yangshan, Guangdong,China | 2 | 1 | 1 | 14 | 1 | 27 | 32 | 1 | 41 |
| [42](http://pubmlst.org/perl/private/bigsdb/bigscurate.pl?page=info&db=pubmlst_csinensis_isolates&id=42) | CSGD11 | Fish, Yangshan, Guangdong,China | 24 | 4 | 1 | 5 | 8 | 15 | 33 | 8 | 42 |
| [43](http://pubmlst.org/perl/private/bigsdb/bigscurate.pl?page=info&db=pubmlst_csinensis_isolates&id=43) | CSGD12 | Fish, Yangshan, Guangdong,China | 1 | 4 | 1 | 3 | 1 | 15 | 34 | 1 | 43 |
| [44](http://pubmlst.org/perl/private/bigsdb/bigscurate.pl?page=info&db=pubmlst_csinensis_isolates&id=44) | CSGD13 | Fish, Yangshan, Guangdong,China | 25 | 1 | 4 | 5 | 9 | 28 | 29 | 2 | 44 |
| [45](http://pubmlst.org/perl/private/bigsdb/bigscurate.pl?page=info&db=pubmlst_csinensis_isolates&id=45) | CSGD14 | Fish, Yangshan, Guangdong,China | 26 | 9 | 1 | 8 | 1 | 29 | 35 | 8 | 45 |
| [46](http://pubmlst.org/perl/private/bigsdb/bigscurate.pl?page=info&db=pubmlst_csinensis_isolates&id=46) | CSGD15 | Fish, Yangshan, Guangdong,China | 8 | 1 | 2 | 8 | 1 | 30 | 36 | 8 | 46 |
| [47](http://pubmlst.org/perl/private/bigsdb/bigscurate.pl?page=info&db=pubmlst_csinensis_isolates&id=47) | CSGD16 | Fish, Yangshan, Guangdong,China | 26 | 9 | 1 | 8 | 1 | 31 | 37 | 1 | 47 |
| [48](http://pubmlst.org/perl/private/bigsdb/bigscurate.pl?page=info&db=pubmlst_csinensis_isolates&id=48) | CSGD17 | Fish, Yangshan, Guangdong,China | 2 | 1 | 1 | 14 | 10 | 6 | 17 | 1 | 48 |
| [49](http://pubmlst.org/perl/private/bigsdb/bigscurate.pl?page=info&db=pubmlst_csinensis_isolates&id=49) | CSGD18 | Fish, Yangshan, Guangdong,China | 6 | 1 | 1 | 5 | 1 | 14 | 38 | 2 | 49 |
| [50](http://pubmlst.org/perl/private/bigsdb/bigscurate.pl?page=info&db=pubmlst_csinensis_isolates&id=50) | CSGD2 | Fish, Yangshan, Guangdong,China | 6 | 1 | 1 | 8 | 8 | 32 | 39 | 1 | 50 |
| [51](http://pubmlst.org/perl/private/bigsdb/bigscurate.pl?page=info&db=pubmlst_csinensis_isolates&id=51) | CSGD3 | Fish, Yangshan, Guangdong,China | 25 | 1 | 1 | 3 | 1 | 33 | 40 | 1 | 51 |
| [52](http://pubmlst.org/perl/private/bigsdb/bigscurate.pl?page=info&db=pubmlst_csinensis_isolates&id=52) | CSGD4 | Fish, Yangshan, Guangdong,China | 27 | 10 | 1 | 1 | 2 | 34 | 41 | 1 | 52 |
| [53](http://pubmlst.org/perl/private/bigsdb/bigscurate.pl?page=info&db=pubmlst_csinensis_isolates&id=53) | CSGD9 | Fish, Yangshan, Guangdong,China | 26 | 4 | 1 | 5 | 1 | 15 | 42 | 1 | 53 |
| [54](http://pubmlst.org/perl/private/bigsdb/bigscurate.pl?page=info&db=pubmlst_csinensis_isolates&id=54) | CSGX1 | Fish, Binyang, Guangxi,China | 25 | 1 | 1 | 3 | 1 | 1 | 43 | 1 | 54 |
| [55](http://pubmlst.org/perl/private/bigsdb/bigscurate.pl?page=info&db=pubmlst_csinensis_isolates&id=55) | CSGX10 | Fish, Binyang, Guangxi,China | 28 | 1 | 1 | 6 | 3 | 4 | 44 | 1 | 55 |
| [56](http://pubmlst.org/perl/private/bigsdb/bigscurate.pl?page=info&db=pubmlst_csinensis_isolates&id=56) | CSGX11 | Fish, Binyang, Guangxi,China | 29 | 9 | 1 | 8 | 1 | 31 | 45 | 8 | 56 |
| [57](http://pubmlst.org/perl/private/bigsdb/bigscurate.pl?page=info&db=pubmlst_csinensis_isolates&id=57) | CSGX12 | Fish, Binyang, Guangxi,China | 30 | 1 | 8 | 4 | 1 | 6 | 17 | 4 | 57 |
| [58](http://pubmlst.org/perl/private/bigsdb/bigscurate.pl?page=info&db=pubmlst_csinensis_isolates&id=58) | CSGX13 | Fish, Binyang, Guangxi,China | 3 | 1 | 1 | 13 | 11 | 1 | 46 | 1 | 58 |
| [59](http://pubmlst.org/perl/private/bigsdb/bigscurate.pl?page=info&db=pubmlst_csinensis_isolates&id=59) | CSGX14 | Fish, Binyang, Guangxi,China | 2 | 7 | 1 | 1 | 12 | 35 | 1 | 1 | 59 |
| [60](http://pubmlst.org/perl/private/bigsdb/bigscurate.pl?page=info&db=pubmlst_csinensis_isolates&id=60) | CSGX15 | Fish, Binyang, Guangxi,China | 32 | 2 | 1 | 8 | 13 | 4 | 47 | 1 | 60 |
| [61](http://pubmlst.org/perl/private/bigsdb/bigscurate.pl?page=info&db=pubmlst_csinensis_isolates&id=61) | CSGX16 | Fish, Binyang, Guangxi,China | 6 | 1 | 1 | 1 | 1 | 36 | 21 | 1 | 61 |
| [62](http://pubmlst.org/perl/private/bigsdb/bigscurate.pl?page=info&db=pubmlst_csinensis_isolates&id=62) | CSGX17 | Fish, Binyang, Guangxi,China | 2 | 1 | 1 | 14 | 10 | 37 | 1 | 1 | 62 |
| [63](http://pubmlst.org/perl/private/bigsdb/bigscurate.pl?page=info&db=pubmlst_csinensis_isolates&id=63) | CSGX18 | Fish, Binyang, Guangxi,China | 33 | 1 | 1 | 3 | 14 | 6 | 48 | 1 | 63 |
| [64](http://pubmlst.org/perl/private/bigsdb/bigscurate.pl?page=info&db=pubmlst_csinensis_isolates&id=64) | CSGX2 | Fish, Binyang, Guangxi,China | 31 | 1 | 9 | 3 | 8 | 38 | 49 | 6 | 64 |
| [65](http://pubmlst.org/perl/private/bigsdb/bigscurate.pl?page=info&db=pubmlst_csinensis_isolates&id=65) | CSGX3 | Fish, Binyang, Guangxi,China | 6 | 1 | 8 | 15 | 1 | 39 | 50 | 1 | 65 |
| [66](http://pubmlst.org/perl/private/bigsdb/bigscurate.pl?page=info&db=pubmlst_csinensis_isolates&id=66) | CSGX4 | Fish, Binyang, Guangxi,China | 34 | 1 | 1 | 16 | 6 | 40 | 51 | 2 | 66 |
| [67](http://pubmlst.org/perl/private/bigsdb/bigscurate.pl?page=info&db=pubmlst_csinensis_isolates&id=67) | CSGX5 | Fish, Binyang, Guangxi,China | 1 | 1 | 1 | 3 | 1 | 41 | 17 | 1 | 67 |
| [68](http://pubmlst.org/perl/private/bigsdb/bigscurate.pl?page=info&db=pubmlst_csinensis_isolates&id=68) | CSGX6 | Fish, Binyang, Guangxi,China | 12 | 1 | 1 | 1 | 9 | 42 | 14 | 1 | 68 |
| [69](http://pubmlst.org/perl/private/bigsdb/bigscurate.pl?page=info&db=pubmlst_csinensis_isolates&id=69) | CSGX7 | Fish, Binyang, Guangxi,China | 31 | 2 | 1 | 3 | 15 | 43 | 10 | 1 | 69 |
| [70](http://pubmlst.org/perl/private/bigsdb/bigscurate.pl?page=info&db=pubmlst_csinensis_isolates&id=70) | CSGX8 | Fish, Binyang, Guangxi,China | 36 | 1 | 1 | 17 | 1 | 44 | 1 | 4 | 70 |
| [71](http://pubmlst.org/perl/private/bigsdb/bigscurate.pl?page=info&db=pubmlst_csinensis_isolates&id=71) | CSGX9 | Fish, Binyang, Guangxi,China | 35 | 4 | 1 | 5 | 1 | 15 | 26 | 1 | 71 |
| [72](http://pubmlst.org/perl/private/bigsdb/bigscurate.pl?page=info&db=pubmlst_csinensis_isolates&id=72) | CSHB1 | Cat, Xiangyang, Hubei, China | 31 | 1 | 1 | 5 | 1 | 34 | 52 | 6 | 72 |
| [73](http://pubmlst.org/perl/private/bigsdb/bigscurate.pl?page=info&db=pubmlst_csinensis_isolates&id=73) | CSHB10 | Cat, Xiangyang, Hubei, China | 37 | 1 | 1 | 3 | 1 | 45 | 53 | 4 | 73 |
| [74](http://pubmlst.org/perl/private/bigsdb/bigscurate.pl?page=info&db=pubmlst_csinensis_isolates&id=74) | CSHB12 | Cat, Xiangyang, Hubei, China | 26 | 1 | 1 | 5 | 16 | 2 | 54 | 4 | 74 |
| [75](http://pubmlst.org/perl/private/bigsdb/bigscurate.pl?page=info&db=pubmlst_csinensis_isolates&id=75) | CSHB15 | Cat, Xiangyang, Hubei, China | 5 | 1 | 1 | 6 | 2 | 6 | 1 | 1 | 75 |
| [76](http://pubmlst.org/perl/private/bigsdb/bigscurate.pl?page=info&db=pubmlst_csinensis_isolates&id=76) | CSHB17 | Cat, Xiangyang, Hubei, China | 1 | 4 | 1 | 2 | 1 | 46 | 18 | 6 | 76 |
| [77](http://pubmlst.org/perl/private/bigsdb/bigscurate.pl?page=info&db=pubmlst_csinensis_isolates&id=77) | CSHB2 | Cat, Xiangyang, Hubei, China | 38 | 1 | 7 | 2 | 2 | 6 | 55 | 9 | 77 |
| [78](http://pubmlst.org/perl/private/bigsdb/bigscurate.pl?page=info&db=pubmlst_csinensis_isolates&id=78) | CSHB20 | Cat, Xiangyang, Hubei, China | 2 | 4 | 1 | 5 | 1 | 47 | 32 | 1 | 78 |
| [79](http://pubmlst.org/perl/private/bigsdb/bigscurate.pl?page=info&db=pubmlst_csinensis_isolates&id=79) | CSHB3 | Cat, Xiangyang, Hubei, China | 22 | 6 | 1 | 3 | 5 | 12 | 14 | 10 | 79 |
| [80](http://pubmlst.org/perl/private/bigsdb/bigscurate.pl?page=info&db=pubmlst_csinensis_isolates&id=80) | CSHB4 | Cat, Xiangyang, Hubei, China | 39 | 1 | 1 | 5 | 16 | 13 | 56 | 11 | 80 |
| [81](http://pubmlst.org/perl/private/bigsdb/bigscurate.pl?page=info&db=pubmlst_csinensis_isolates&id=81) | CSHB5 | Cat, Xiangyang, Hubei, China | 40 | 2 | 10 | 5 | 1 | 48 | 10 | 6 | 81 |
| [82](http://pubmlst.org/perl/private/bigsdb/bigscurate.pl?page=info&db=pubmlst_csinensis_isolates&id=82) | CSHB6 | Cat, Xiangyang, Hubei, China | 41 | 4 | 1 | 18 | 3 | 49 | 57 | 6 | 82 |
| [83](http://pubmlst.org/perl/private/bigsdb/bigscurate.pl?page=info&db=pubmlst_csinensis_isolates&id=83) | CSHB7 | Cat, Xiangyang, Hubei, China | 6 | 4 | 1 | 5 | 1 | 1 | 14 | 6 | 83 |
| [84](http://pubmlst.org/perl/private/bigsdb/bigscurate.pl?page=info&db=pubmlst_csinensis_isolates&id=84) | CSHB8 | Cat, Xiangyang, Hubei, China | 8 | 1 | 1 | 1 | 16 | 34 | 14 | 4 | 84 |
| [85](http://pubmlst.org/perl/private/bigsdb/bigscurate.pl?page=info&db=pubmlst_csinensis_isolates&id=85) | CSHB9 | Cat, Xiangyang, Hubei, China | 8 | 1 | 1 | 2 | 17 | 50 | 14 | 6 | 85 |
| [86](http://pubmlst.org/perl/private/bigsdb/bigscurate.pl?page=info&db=pubmlst_csinensis_isolates&id=86) | CSHEB1 | Cat, Langfang,Hebei, China | 27 | 8 | 11 | 9 | 1 | 51 | 58 | 6 | 86 |
| [87](http://pubmlst.org/perl/private/bigsdb/bigscurate.pl?page=info&db=pubmlst_csinensis_isolates&id=87) | CSHEB10 | Cat, Langfang,Hebei, China | 42 | 4 | 1 | 5 | 1 | 52 | 1 | 1 | 87 |
| [88](http://pubmlst.org/perl/private/bigsdb/bigscurate.pl?page=info&db=pubmlst_csinensis_isolates&id=88) | CSHEB11 | Cat, Langfang,Hebei, China | 27 | 4 | 1 | 5 | 1 | 53 | 59 | 6 | 88 |
| [89](http://pubmlst.org/perl/private/bigsdb/bigscurate.pl?page=info&db=pubmlst_csinensis_isolates&id=89) | CSHEB12 | Cat, Langfang,Hebei, China | 43 | 8 | 4 | 19 | 1 | 54 | 60 | 1 | 89 |
| [90](http://pubmlst.org/perl/private/bigsdb/bigscurate.pl?page=info&db=pubmlst_csinensis_isolates&id=90) | CSHEB15 | Cat, Langfang,Hebei, China | 27 | 4 | 1 | 3 | 1 | 15 | 59 | 6 | 90 |
| [91](http://pubmlst.org/perl/private/bigsdb/bigscurate.pl?page=info&db=pubmlst_csinensis_isolates&id=91) | CSHEB16 | Cat, Langfang,Hebei, China | 44 | 1 | 1 | 5 | 1 | 13 | 53 | 8 | 91 |
| [92](http://pubmlst.org/perl/private/bigsdb/bigscurate.pl?page=info&db=pubmlst_csinensis_isolates&id=92) | CSHEB17 | Cat, Langfang,Hebei, China | 5 | 1 | 1 | 6 | 2 | 55 | 61 | 4 | 92 |
| [93](http://pubmlst.org/perl/private/bigsdb/bigscurate.pl?page=info&db=pubmlst_csinensis_isolates&id=93) | CSHEB18 | Cat, Langfang,Hebei, China | 2 | 1 | 1 | 8 | 18 | 10 | 57 | 6 | 93 |
| [94](http://pubmlst.org/perl/private/bigsdb/bigscurate.pl?page=info&db=pubmlst_csinensis_isolates&id=94) | CSHEB2 | Cat, Langfang,Hebei, China | 34 | 8 | 4 | 20 | 1 | 54 | 62 | 5 | 94 |
| [95](http://pubmlst.org/perl/private/bigsdb/bigscurate.pl?page=info&db=pubmlst_csinensis_isolates&id=95) | CSHEB3 | Cat, Langfang,Hebei, China | 45 | 1 | 1 | 2 | 1 | 13 | 53 | 1 | 95 |
| [96](http://pubmlst.org/perl/private/bigsdb/bigscurate.pl?page=info&db=pubmlst_csinensis_isolates&id=96) | CSHEB4 | Cat, Langfang,Hebei, China | 6 | 8 | 4 | 8 | 1 | 54 | 60 | 1 | 96 |
| [97](http://pubmlst.org/perl/private/bigsdb/bigscurate.pl?page=info&db=pubmlst_csinensis_isolates&id=97) | CSHEB5 | Cat, Langfang,Hebei, China | 46 | 4 | 1 | 6 | 1 | 11 | 63 | 2 | 97 |
| [98](http://pubmlst.org/perl/private/bigsdb/bigscurate.pl?page=info&db=pubmlst_csinensis_isolates&id=98) | CSHEB6 | Cat, Langfang,Hebei, China | 47 | 1 | 1 | 6 | 3 | 10 | 64 | 1 | 98 |
| [99](http://pubmlst.org/perl/private/bigsdb/bigscurate.pl?page=info&db=pubmlst_csinensis_isolates&id=99) | CSHEB7 | Cat, Langfang,Hebei, China | 6 | 9 | 1 | 2 | 3 | 56 | 1 | 1 | 99 |
| [100](http://pubmlst.org/perl/private/bigsdb/bigscurate.pl?page=info&db=pubmlst_csinensis_isolates&id=100) | CSHEB8 | Cat, Langfang,Hebei, China | 41 | 1 | 12 | 5 | 1 | 10 | 14 | 1 | 100 |
| [101](http://pubmlst.org/perl/private/bigsdb/bigscurate.pl?page=info&db=pubmlst_csinensis_isolates&id=101) | CSHEB9 | Cat, Langfang,Hebei, China | 40 | 1 | 1 | 8 | 19 | 25 | 65 | 1 | 101 |
| [102](http://pubmlst.org/perl/private/bigsdb/bigscurate.pl?page=info&db=pubmlst_csinensis_isolates&id=102) | CSHLJ1 | Dog, Zhaoyuan,Heilongjiang,China | 23 | 1 | 1 | 11 | 2 | 43 | 1 | 1 | 102 |
| [103](http://pubmlst.org/perl/private/bigsdb/bigscurate.pl?page=info&db=pubmlst_csinensis_isolates&id=103) | CSHLJ10 | Dog, Zhaoyuan,Heilongjiang,China | 48 | 1 | 1 | 8 | 1 | 13 | 66 | 6 | 103 |
| [104](http://pubmlst.org/perl/private/bigsdb/bigscurate.pl?page=info&db=pubmlst_csinensis_isolates&id=104) | CSHLJ11 | Dog, Zhaoyuan,Heilongjiang,China | 49 | 1 | 1 | 9 | 1 | 4 | 14 | 1 | 104 |
| [105](http://pubmlst.org/perl/private/bigsdb/bigscurate.pl?page=info&db=pubmlst_csinensis_isolates&id=105) | CSHLJ12 | Dog, Zhaoyuan,Heilongjiang,China | 8 | 10 | 1 | 8 | 1 | 57 | 1 | 1 | 105 |
| [106](http://pubmlst.org/perl/private/bigsdb/bigscurate.pl?page=info&db=pubmlst_csinensis_isolates&id=106) | CSHLJ13 | Dog, Zhaoyuan,Heilongjiang,China | 50 | 1 | 1 | 5 | 1 | 14 | 67 | 6 | 106 |
| [107](http://pubmlst.org/perl/private/bigsdb/bigscurate.pl?page=info&db=pubmlst_csinensis_isolates&id=107) | CSHLJ14 | Dog, Zhaoyuan,Heilongjiang,China | 44 | 1 | 1 | 8 | 2 | 58 | 68 | 1 | 107 |
| [108](http://pubmlst.org/perl/private/bigsdb/bigscurate.pl?page=info&db=pubmlst_csinensis_isolates&id=108) | CSHLJ15 | Dog, Zhaoyuan,Heilongjiang,China | 51 | 6 | 1 | 5 | 1 | 12 | 1 | 8 | 108 |
| [109](http://pubmlst.org/perl/private/bigsdb/bigscurate.pl?page=info&db=pubmlst_csinensis_isolates&id=109) | CSHLJ16 | Dog, Zhaoyuan,Heilongjiang,China | 52 | 1 | 1 | 5 | 1 | 44 | 69 | 2 | 109 |
| [110](http://pubmlst.org/perl/private/bigsdb/bigscurate.pl?page=info&db=pubmlst_csinensis_isolates&id=110) | CSHLJ2 | Dog, Zhaoyuan,Heilongjiang,China | 6 | 1 | 1 | 13 | 1 | 59 | 70 | 1 | 110 |
| [111](http://pubmlst.org/perl/private/bigsdb/bigscurate.pl?page=info&db=pubmlst_csinensis_isolates&id=111) | CSHLJ3 | Dog, Zhaoyuan,Heilongjiang,China | 51 | 1 | 1 | 2 | 1 | 60 | 1 | 6 | 111 |
| [112](http://pubmlst.org/perl/private/bigsdb/bigscurate.pl?page=info&db=pubmlst_csinensis_isolates&id=112) | CSHLJ4 | Dog, Zhaoyuan,Heilongjiang,China | 53 | 1 | 1 | 13 | 1 | 4 | 1 | 1 | 112 |
| [113](http://pubmlst.org/perl/private/bigsdb/bigscurate.pl?page=info&db=pubmlst_csinensis_isolates&id=113) | CSHLJ5 | Dog, Zhaoyuan,Heilongjiang,China | 51 | 1 | 11 | 21 | 1 | 61 | 71 | 1 | 113 |
| [114](http://pubmlst.org/perl/private/bigsdb/bigscurate.pl?page=info&db=pubmlst_csinensis_isolates&id=114) | CSHLJ6 | Dog, Zhaoyuan,Heilongjiang,China | 55 | 6 | 1 | 21 | 1 | 62 | 25 | 2 | 114 |
| [115](http://pubmlst.org/perl/private/bigsdb/bigscurate.pl?page=info&db=pubmlst_csinensis_isolates&id=115) | CSHLJ7 | Dog, Zhaoyuan,Heilongjiang,China | 48 | 8 | 11 | 6 | 1 | 63 | 70 | 1 | 115 |
| [116](http://pubmlst.org/perl/private/bigsdb/bigscurate.pl?page=info&db=pubmlst_csinensis_isolates&id=116) | CSHLJ8 | Dog, Zhaoyuan,Heilongjiang,China | 5 | 1 | 11 | 8 | 1 | 61 | 58 | 1 | 116 |
| [117](http://pubmlst.org/perl/private/bigsdb/bigscurate.pl?page=info&db=pubmlst_csinensis_isolates&id=117) | CSHLJ9 | Dog, Zhaoyuan,Heilongjiang,China | 3 | 6 | 1 | 5 | 1 | 64 | 72 | 1 | 117 |
| [118](http://pubmlst.org/perl/private/bigsdb/bigscurate.pl?page=info&db=pubmlst_csinensis_isolates&id=118) | CSHN1 | Cat,Xinyang,Henan, China | 31 | 4 | 1 | 5 | 20 | 65 | 1 | 1 | 118 |
| [119](http://pubmlst.org/perl/private/bigsdb/bigscurate.pl?page=info&db=pubmlst_csinensis_isolates&id=119) | CSHN10 | Cat,Xinyang,Henan, China | 37 | 1 | 1 | 5 | 20 | 66 | 73 | 6 | 119 |
| [120](http://pubmlst.org/perl/private/bigsdb/bigscurate.pl?page=info&db=pubmlst_csinensis_isolates&id=120) | CSHN11 | Cat,Xinyang,Henan, China | 2 | 1 | 2 | 6 | 20 | 17 | 20 | 8 | 120 |
| [121](http://pubmlst.org/perl/private/bigsdb/bigscurate.pl?page=info&db=pubmlst_csinensis_isolates&id=121) | CSHN12 | Cat,Xinyang,Henan, China | 1 | 1 | 1 | 5 | 1 | 6 | 1 | 1 | 121 |
| [122](http://pubmlst.org/perl/private/bigsdb/bigscurate.pl?page=info&db=pubmlst_csinensis_isolates&id=122) | CSHN13 | Cat,Xinyang,Henan, China | 10 | 6 | 1 | 6 | 1 | 67 | 74 | 12 | 122 |
| [123](http://pubmlst.org/perl/private/bigsdb/bigscurate.pl?page=info&db=pubmlst_csinensis_isolates&id=123) | CSHN14 | Cat,Xinyang,Henan, China | 8 | 1 | 1 | 22 | 21 | 50 | 1 | 6 | 123 |
| [124](http://pubmlst.org/perl/private/bigsdb/bigscurate.pl?page=info&db=pubmlst_csinensis_isolates&id=124) | CSHN15 | Cat,Xinyang,Henan, China | 2 | 1 | 1 | 5 | 5 | 33 | 75 | 6 | 124 |
| [125](http://pubmlst.org/perl/private/bigsdb/bigscurate.pl?page=info&db=pubmlst_csinensis_isolates&id=125) | CSHN16 | Cat,Xinyang,Henan, China | 5 | 1 | 1 | 5 | 1 | 34 | 14 | 1 | 125 |
| [126](http://pubmlst.org/perl/private/bigsdb/bigscurate.pl?page=info&db=pubmlst_csinensis_isolates&id=126) | CSHN17 | Cat,Xinyang,Henan, China | 40 | 3 | 1 | 2 | 22 | 10 | 57 | 6 | 126 |
| [127](http://pubmlst.org/perl/private/bigsdb/bigscurate.pl?page=info&db=pubmlst_csinensis_isolates&id=127) | CSHN18 | Cat,Xinyang,Henan, China | 6 | 1 | 1 | 8 | 21 | 10 | 1 | 6 | 127 |
| [128](http://pubmlst.org/perl/private/bigsdb/bigscurate.pl?page=info&db=pubmlst_csinensis_isolates&id=128) | CSHN19 | Cat,Xinyang,Henan, China | 18 | 4 | 1 | 5 | 21 | 25 | 28 | 13 | 128 |
| [129](http://pubmlst.org/perl/private/bigsdb/bigscurate.pl?page=info&db=pubmlst_csinensis_isolates&id=129) | CSHN2 | Cat,Xinyang,Henan, China | 1 | 2 | 1 | 3 | 1 | 4 | 76 | 6 | 129 |
| [130](http://pubmlst.org/perl/private/bigsdb/bigscurate.pl?page=info&db=pubmlst_csinensis_isolates&id=130) | CSHN20 | Cat,Xinyang,Henan, China | 2 | 1 | 1 | 5 | 22 | 6 | 77 | 4 | 130 |
| [131](http://pubmlst.org/perl/private/bigsdb/bigscurate.pl?page=info&db=pubmlst_csinensis_isolates&id=131) | CSHN3 | Cat,Xinyang,Henan, China | 25 | 4 | 1 | 1 | 10 | 68 | 1 | 13 | 131 |
| [132](http://pubmlst.org/perl/private/bigsdb/bigscurate.pl?page=info&db=pubmlst_csinensis_isolates&id=132) | CSHN4 | Cat,Xinyang,Henan, China | 25 | 4 | 13 | 16 | 5 | 25 | 78 | 1 | 132 |
| [133](http://pubmlst.org/perl/private/bigsdb/bigscurate.pl?page=info&db=pubmlst_csinensis_isolates&id=133) | CSHN5 | Cat,Xinyang,Henan, China | 25 | 1 | 1 | 2 | 1 | 10 | 57 | 1 | 133 |
| [134](http://pubmlst.org/perl/private/bigsdb/bigscurate.pl?page=info&db=pubmlst_csinensis_isolates&id=134) | CSHN6 | Cat,Xinyang,Henan, China | 56 | 4 | 14 | 5 | 1 | 15 | 79 | 6 | 134 |
| [135](http://pubmlst.org/perl/private/bigsdb/bigscurate.pl?page=info&db=pubmlst_csinensis_isolates&id=135) | CSHN7 | Cat,Xinyang,Henan, China | 2 | 4 | 13 | 9 | 1 | 25 | 28 | 6 | 135 |
| [136](http://pubmlst.org/perl/private/bigsdb/bigscurate.pl?page=info&db=pubmlst_csinensis_isolates&id=136) | CSHN8 | Cat,Xinyang,Henan, China | 1 | 2 | 1 | 6 | 3 | 69 | 10 | 1 | 136 |
| [137](http://pubmlst.org/perl/private/bigsdb/bigscurate.pl?page=info&db=pubmlst_csinensis_isolates&id=137) | CSHN9 | Cat,Xinyang,Henan, China | 2 | 4 | 14 | 5 | 1 | 15 | 79 | 4 | 137 |
| [138](http://pubmlst.org/perl/private/bigsdb/bigscurate.pl?page=info&db=pubmlst_csinensis_isolates&id=138) | CSHUN1 | Cat, Yueyang, Hunan, China | 23 | 1 | 1 | 1 | 2 | 34 | 78 | 6 | 138 |
| [139](http://pubmlst.org/perl/private/bigsdb/bigscurate.pl?page=info&db=pubmlst_csinensis_isolates&id=139) | CSHUN10 | Cat, Yueyang, Hunan, China | 44 | 1 | 1 | 6 | 1 | 70 | 14 | 6 | 139 |
| [140](http://pubmlst.org/perl/private/bigsdb/bigscurate.pl?page=info&db=pubmlst_csinensis_isolates&id=140) | CSHUN11 | Cat, Yueyang, Hunan, China | 57 | 8 | 4 | 5 | 1 | 54 | 60 | 1 | 140 |
| [141](http://pubmlst.org/perl/private/bigsdb/bigscurate.pl?page=info&db=pubmlst_csinensis_isolates&id=141) | CSHUN12 | Cat, Yueyang, Hunan, China | 13 | 9 | 1 | 6 | 2 | 71 | 1 | 6 | 141 |
| [142](http://pubmlst.org/perl/private/bigsdb/bigscurate.pl?page=info&db=pubmlst_csinensis_isolates&id=142) | CSHUN13 | Cat, Yueyang, Hunan, China | 58 | 4 | 1 | 5 | 1 | 72 | 1 | 6 | 142 |
| [143](http://pubmlst.org/perl/private/bigsdb/bigscurate.pl?page=info&db=pubmlst_csinensis_isolates&id=143) | CSHUN14 | Cat, Yueyang, Hunan, China | 58 | 4 | 1 | 5 | 1 | 72 | 15 | 6 | 143 |
| [144](http://pubmlst.org/perl/private/bigsdb/bigscurate.pl?page=info&db=pubmlst_csinensis_isolates&id=144) | CSHUN15 | Cat, Yueyang, Hunan, China | 13 | 4 | 1 | 5 | 1 | 73 | 80 | 4 | 144 |
| [145](http://pubmlst.org/perl/private/bigsdb/bigscurate.pl?page=info&db=pubmlst_csinensis_isolates&id=145) | CSHUN16 | Cat, Yueyang, Hunan, China | 27 | 1 | 1 | 5 | 1 | 74 | 81 | 1 | 145 |
| [146](http://pubmlst.org/perl/private/bigsdb/bigscurate.pl?page=info&db=pubmlst_csinensis_isolates&id=146) | CSHUN17 | Cat, Yueyang, Hunan, China | 15 | 1 | 1 | 2 | 1 | 75 | 1 | 6 | 146 |
| [147](http://pubmlst.org/perl/private/bigsdb/bigscurate.pl?page=info&db=pubmlst_csinensis_isolates&id=147) | CSHUN18 | Cat, Yueyang, Hunan, China | 59 | 4 | 1 | 3 | 1 | 76 | 65 | 4 | 147 |
| [148](http://pubmlst.org/perl/private/bigsdb/bigscurate.pl?page=info&db=pubmlst_csinensis_isolates&id=148) | CSHUN2 | Cat, Yueyang, Hunan, China | 8 | 11 | 1 | 5 | 3 | 1 | 82 | 6 | 148 |
| [149](http://pubmlst.org/perl/private/bigsdb/bigscurate.pl?page=info&db=pubmlst_csinensis_isolates&id=149) | CSHUN3 | Cat, Yueyang, Hunan, China | 25 | 1 | 1 | 5 | 3 | 6 | 1 | 6 | 149 |
| [150](http://pubmlst.org/perl/private/bigsdb/bigscurate.pl?page=info&db=pubmlst_csinensis_isolates&id=150) | CSHUN4 | Cat, Yueyang, Hunan, China | 3 | 1 | 1 | 2 | 1 | 43 | 1 | 1 | 150 |
| [151](http://pubmlst.org/perl/private/bigsdb/bigscurate.pl?page=info&db=pubmlst_csinensis_isolates&id=151) | CSHUN5 | Cat, Yueyang, Hunan, China | 6 | 1 | 1 | 1 | 9 | 34 | 14 | 6 | 151 |
| [152](http://pubmlst.org/perl/private/bigsdb/bigscurate.pl?page=info&db=pubmlst_csinensis_isolates&id=152) | CSHUN6 | Cat, Yueyang, Hunan, China | 26 | 10 | 1 | 2 | 1 | 4 | 1 | 6 | 152 |
| [153](http://pubmlst.org/perl/private/bigsdb/bigscurate.pl?page=info&db=pubmlst_csinensis_isolates&id=153) | CSHUN7 | Cat, Yueyang, Hunan, China | 10 | 1 | 1 | 1 | 1 | 4 | 83 | 1 | 153 |
| [154](http://pubmlst.org/perl/private/bigsdb/bigscurate.pl?page=info&db=pubmlst_csinensis_isolates&id=154) | CSHUN8 | Cat, Yueyang, Hunan, China | 31 | 4 | 1 | 2 | 1 | 15 | 28 | 6 | 154 |
| [155](http://pubmlst.org/perl/private/bigsdb/bigscurate.pl?page=info&db=pubmlst_csinensis_isolates&id=155) | CSHUN9 | Cat, Yueyang, Hunan, China | 1 | 9 | 1 | 1 | 5 | 77 | 1 | 4 | 155 |
| [156](http://pubmlst.org/perl/private/bigsdb/bigscurate.pl?page=info&db=pubmlst_csinensis_isolates&id=156) | CSJL1 | Dog, Changchun, Jilin, China | 8 | 1 | 1 | 5 | 1 | 6 | 1 | 6 | 156 |
| [157](http://pubmlst.org/perl/private/bigsdb/bigscurate.pl?page=info&db=pubmlst_csinensis_isolates&id=157) | CSJL10 | Dog, Changchun, Jilin, China | 6 | 1 | 4 | 5 | 1 | 40 | 20 | 1 | 157 |
| [158](http://pubmlst.org/perl/private/bigsdb/bigscurate.pl?page=info&db=pubmlst_csinensis_isolates&id=158) | CSJL11 | Dog, Changchun, Jilin, China | 3 | 1 | 11 | 9 | 2 | 78 | 58 | 1 | 158 |
| [159](http://pubmlst.org/perl/private/bigsdb/bigscurate.pl?page=info&db=pubmlst_csinensis_isolates&id=159) | CSJL12 | Dog, Changchun, Jilin, China | 8 | 1 | 1 | 8 | 1 | 13 | 84 | 1 | 159 |
| [160](http://pubmlst.org/perl/private/bigsdb/bigscurate.pl?page=info&db=pubmlst_csinensis_isolates&id=160) | CSJL13 | Dog, Changchun, Jilin, China | 60 | 1 | 1 | 5 | 1 | 6 | 85 | 1 | 160 |
| [161](http://pubmlst.org/perl/private/bigsdb/bigscurate.pl?page=info&db=pubmlst_csinensis_isolates&id=161) | CSJL14 | Dog, Changchun, Jilin, China | 25 | 1 | 1 | 23 | 1 | 57 | 86 | 1 | 161 |
| [162](http://pubmlst.org/perl/private/bigsdb/bigscurate.pl?page=info&db=pubmlst_csinensis_isolates&id=162) | CSJL15 | Dog, Changchun, Jilin, China | 19 | 1 | 1 | 3 | 3 | 26 | 87 | 4 | 162 |
| [163](http://pubmlst.org/perl/private/bigsdb/bigscurate.pl?page=info&db=pubmlst_csinensis_isolates&id=163) | CSJL16 | Dog, Changchun, Jilin, China | 8 | 6 | 1 | 24 | 1 | 24 | 88 | 6 | 163 |
| [164](http://pubmlst.org/perl/private/bigsdb/bigscurate.pl?page=info&db=pubmlst_csinensis_isolates&id=164) | CSJL17 | Dog, Changchun, Jilin, China | 27 | 1 | 1 | 2 | 1 | 6 | 72 | 1 | 164 |
| [165](http://pubmlst.org/perl/private/bigsdb/bigscurate.pl?page=info&db=pubmlst_csinensis_isolates&id=165) | CSJL18 | Dog, Changchun, Jilin, China | 10 | 4 | 5 | 5 | 1 | 15 | 89 | 6 | 165 |
| [166](http://pubmlst.org/perl/private/bigsdb/bigscurate.pl?page=info&db=pubmlst_csinensis_isolates&id=166) | CSJL19 | Dog, Changchun, Jilin, China | 6 | 1 | 1 | 1 | 1 | 4 | 90 | 6 | 166 |
| [167](http://pubmlst.org/perl/private/bigsdb/bigscurate.pl?page=info&db=pubmlst_csinensis_isolates&id=167) | CSJL2 | Dog, Changchun, Jilin, China | 6 | 1 | 1 | 3 | 1 | 33 | 1 | 1 | 167 |
| [168](http://pubmlst.org/perl/private/bigsdb/bigscurate.pl?page=info&db=pubmlst_csinensis_isolates&id=168) | CSJL3 | Dog, Changchun, Jilin, China | 8 | 1 | 1 | 8 | 1 | 33 | 57 | 1 | 168 |
| [169](http://pubmlst.org/perl/private/bigsdb/bigscurate.pl?page=info&db=pubmlst_csinensis_isolates&id=169) | CSJL5 | Dog, Changchun, Jilin, China | 61 | 8 | 4 | 5 | 1 | 20 | 60 | 1 | 169 |
| [170](http://pubmlst.org/perl/private/bigsdb/bigscurate.pl?page=info&db=pubmlst_csinensis_isolates&id=170) | CSJL7 | Dog, Changchun, Jilin, China | 25 | 1 | 1 | 10 | 1 | 34 | 81 | 6 | 170 |
| [171](http://pubmlst.org/perl/private/bigsdb/bigscurate.pl?page=info&db=pubmlst_csinensis_isolates&id=171) | CSJL8 | Dog, Changchun, Jilin, China | 62 | 1 | 1 | 6 | 1 | 1 | 91 | 6 | 171 |
| [172](http://pubmlst.org/perl/private/bigsdb/bigscurate.pl?page=info&db=pubmlst_csinensis_isolates&id=172) | CSJL9 | Dog, Changchun, Jilin, China | 31 | 4 | 1 | 6 | 1 | 79 | 92 | 6 | 172 |
| [173](http://pubmlst.org/perl/private/bigsdb/bigscurate.pl?page=info&db=pubmlst_csinensis_isolates&id=173) | CSJS1 | Cat, Nanjing, Jiangsu, China | 6 | 1 | 1 | 1 | 1 | 43 | 93 | 1 | 173 |
| [174](http://pubmlst.org/perl/private/bigsdb/bigscurate.pl?page=info&db=pubmlst_csinensis_isolates&id=174) | CSJS10 | Cat, Nanjing, Jiangsu, China | 25 | 4 | 1 | 5 | 1 | 80 | 28 | 1 | 174 |
| [175](http://pubmlst.org/perl/private/bigsdb/bigscurate.pl?page=info&db=pubmlst_csinensis_isolates&id=175) | CSJS11 | Cat, Nanjing, Jiangsu, China | 2 | 1 | 15 | 8 | 1 | 3 | 14 | 1 | 175 |
| [176](http://pubmlst.org/perl/private/bigsdb/bigscurate.pl?page=info&db=pubmlst_csinensis_isolates&id=176) | CSJS12 | Cat, Nanjing, Jiangsu, China | 15 | 2 | 1 | 5 | 1 | 81 | 10 | 14 | 176 |
| [177](http://pubmlst.org/perl/private/bigsdb/bigscurate.pl?page=info&db=pubmlst_csinensis_isolates&id=177) | CSJS13 | Cat, Nanjing, Jiangsu, China | 15 | 2 | 1 | 5 | 1 | 81 | 10 | 14 | 176 |
| [178](http://pubmlst.org/perl/private/bigsdb/bigscurate.pl?page=info&db=pubmlst_csinensis_isolates&id=178) | CSJS16 | Cat, Nanjing, Jiangsu, China | 31 | 2 | 1 | 5 | 3 | 4 | 94 | 1 | 177 |
| [179](http://pubmlst.org/perl/private/bigsdb/bigscurate.pl?page=info&db=pubmlst_csinensis_isolates&id=179) | CSJS2 | Cat, Nanjing, Jiangsu, China | 27 | 1 | 11 | 1 | 1 | 34 | 95 | 1 | 178 |
| [180](http://pubmlst.org/perl/private/bigsdb/bigscurate.pl?page=info&db=pubmlst_csinensis_isolates&id=180) | CSJS3 | Cat, Nanjing, Jiangsu, China | 23 | 1 | 1 | 8 | 1 | 82 | 96 | 1 | 179 |
| [181](http://pubmlst.org/perl/private/bigsdb/bigscurate.pl?page=info&db=pubmlst_csinensis_isolates&id=181) | CSJS4 | Cat, Nanjing, Jiangsu, China | 10 | 1 | 1 | 3 | 6 | 1 | 14 | 1 | 180 |
| [182](http://pubmlst.org/perl/private/bigsdb/bigscurate.pl?page=info&db=pubmlst_csinensis_isolates&id=182) | CSJS6 | Cat, Nanjing, Jiangsu, China | 12 | 4 | 1 | 8 | 1 | 53 | 59 | 1 | 181 |
| [183](http://pubmlst.org/perl/private/bigsdb/bigscurate.pl?page=info&db=pubmlst_csinensis_isolates&id=183) | CSJS7 | Cat, Nanjing, Jiangsu, China | 52 | 4 | 1 | 14 | 2 | 80 | 18 | 2 | 182 |
| [184](http://pubmlst.org/perl/private/bigsdb/bigscurate.pl?page=info&db=pubmlst_csinensis_isolates&id=184) | CSJS8 | Cat, Nanjing, Jiangsu, China | 2 | 2 | 4 | 2 | 3 | 17 | 97 | 2 | 183 |
| [185](http://pubmlst.org/perl/private/bigsdb/bigscurate.pl?page=info&db=pubmlst_csinensis_isolates&id=185) | CSJX1 | Cat, Jiujiang, Jiangxi, China | 12 | 4 | 1 | 2 | 1 | 15 | 98 | 6 | 184 |
| [186](http://pubmlst.org/perl/private/bigsdb/bigscurate.pl?page=info&db=pubmlst_csinensis_isolates&id=186) | CSJX10 | Cat, Jiujiang, Jiangxi, China | 63 | 1 | 1 | 23 | 5 | 83 | 1 | 7 | 185 |
| [187](http://pubmlst.org/perl/private/bigsdb/bigscurate.pl?page=info&db=pubmlst_csinensis_isolates&id=187) | CSJX11 | Cat, Jiujiang, Jiangxi, China | 23 | 1 | 1 | 3 | 23 | 18 | 99 | 1 | 186 |
| [188](http://pubmlst.org/perl/private/bigsdb/bigscurate.pl?page=info&db=pubmlst_csinensis_isolates&id=188) | CSJX13 | Cat, Jiujiang, Jiangxi, China | 44 | 4 | 1 | 25 | 1 | 84 | 26 | 1 | 187 |
| [189](http://pubmlst.org/perl/private/bigsdb/bigscurate.pl?page=info&db=pubmlst_csinensis_isolates&id=189) | CSJX14 | Cat, Jiujiang, Jiangxi, China | 64 | 1 | 1 | 8 | 24 | 85 | 100 | 4 | 188 |
| [190](http://pubmlst.org/perl/private/bigsdb/bigscurate.pl?page=info&db=pubmlst_csinensis_isolates&id=190) | CSJX15 | Cat, Jiujiang, Jiangxi, China | 5 | 4 | 16 | 5 | 5 | 16 | 18 | 7 | 189 |
| [191](http://pubmlst.org/perl/private/bigsdb/bigscurate.pl?page=info&db=pubmlst_csinensis_isolates&id=191) | CSJX16 | Cat, Jiujiang, Jiangxi, China | 5 | 10 | 12 | 5 | 5 | 4 | 101 | 6 | 190 |
| [192](http://pubmlst.org/perl/private/bigsdb/bigscurate.pl?page=info&db=pubmlst_csinensis_isolates&id=192) | CSJX2 | Cat, Jiujiang, Jiangxi, China | 2 | 4 | 1 | 1 | 1 | 4 | 82 | 6 | 191 |
| [193](http://pubmlst.org/perl/private/bigsdb/bigscurate.pl?page=info&db=pubmlst_csinensis_isolates&id=193) | CSJX3 | Cat, Jiujiang, Jiangxi, China | 65 | 1 | 1 | 5 | 1 | 1 | 39 | 4 | 192 |
| [194](http://pubmlst.org/perl/private/bigsdb/bigscurate.pl?page=info&db=pubmlst_csinensis_isolates&id=194) | CSJX4 | Cat, Jiujiang, Jiangxi, China | 31 | 1 | 1 | 2 | 1 | 86 | 1 | 6 | 193 |
| [195](http://pubmlst.org/perl/private/bigsdb/bigscurate.pl?page=info&db=pubmlst_csinensis_isolates&id=195) | CSJX5 | Cat, Jiujiang, Jiangxi, China | 14 | 1 | 1 | 1 | 5 | 4 | 102 | 1 | 194 |
| [196](http://pubmlst.org/perl/private/bigsdb/bigscurate.pl?page=info&db=pubmlst_csinensis_isolates&id=196) | CSJX7 | Cat, Jiujiang, Jiangxi, China | 66 | 6 | 7 | 3 | 25 | 12 | 1 | 4 | 195 |
| [197](http://pubmlst.org/perl/private/bigsdb/bigscurate.pl?page=info&db=pubmlst_csinensis_isolates&id=197) | CSJX8 | Cat, Jiujiang, Jiangxi, China | 44 | 4 | 1 | 3 | 2 | 87 | 103 | 1 | 196 |
| [198](http://pubmlst.org/perl/private/bigsdb/bigscurate.pl?page=info&db=pubmlst_csinensis_isolates&id=198) | CSJX9 | Cat, Jiujiang, Jiangxi, China | 31 | 1 | 1 | 5 | 3 | 88 | 1 | 1 | 197 |
| [199](http://pubmlst.org/perl/private/bigsdb/bigscurate.pl?page=info&db=pubmlst_csinensis_isolates&id=199) | CSSC10 | Cat, Chongqing, Sichuan, China | 42 | 1 | 1 | 26 | 1 | 6 | 27 | 1 | 198 |
| [200](http://pubmlst.org/perl/private/bigsdb/bigscurate.pl?page=info&db=pubmlst_csinensis_isolates&id=200) | CSSC11 | Cat, Chongqing, Sichuan, China | 67 | 6 | 1 | 5 | 3 | 12 | 14 | 1 | 199 |
| [201](http://pubmlst.org/perl/private/bigsdb/bigscurate.pl?page=info&db=pubmlst_csinensis_isolates&id=201) | CSSC12 | Cat, Chongqing, Sichuan, China | 68 | 12 | 8 | 5 | 26 | 28 | 104 | 1 | 200 |
| [202](http://pubmlst.org/perl/private/bigsdb/bigscurate.pl?page=info&db=pubmlst_csinensis_isolates&id=202) | CSSC13 | Cat, Chongqing, Sichuan, China | 1 | 1 | 1 | 2 | 1 | 27 | 1 | 6 | 201 |
| [203](http://pubmlst.org/perl/private/bigsdb/bigscurate.pl?page=info&db=pubmlst_csinensis_isolates&id=203) | CSSC14 | Cat, Chongqing, Sichuan, China | 23 | 4 | 1 | 5 | 1 | 89 | 105 | 15 | 202 |
| [204](http://pubmlst.org/perl/private/bigsdb/bigscurate.pl?page=info&db=pubmlst_csinensis_isolates&id=204) | CSSC15 | Cat, Chongqing, Sichuan, China | 69 | 1 | 1 | 3 | 1 | 13 | 4 | 15 | 203 |
| [205](http://pubmlst.org/perl/private/bigsdb/bigscurate.pl?page=info&db=pubmlst_csinensis_isolates&id=205) | CSSC16 | Cat, Chongqing, Sichuan, China | 31 | 1 | 1 | 5 | 1 | 6 | 77 | 1 | 204 |
| [206](http://pubmlst.org/perl/private/bigsdb/bigscurate.pl?page=info&db=pubmlst_csinensis_isolates&id=206) | CSSC4 | Cat, Chongqing, Sichuan, China | 70 | 1 | 1 | 8 | 3 | 10 | 57 | 1 | 205 |
| [207](http://pubmlst.org/perl/private/bigsdb/bigscurate.pl?page=info&db=pubmlst_csinensis_isolates&id=207) | CSSC5 | Cat, Chongqing, Sichuan, China | 66 | 4 | 1 | 5 | 23 | 15 | 28 | 6 | 206 |
| [208](http://pubmlst.org/perl/private/bigsdb/bigscurate.pl?page=info&db=pubmlst_csinensis_isolates&id=208) | CSSC6 | Cat, Chongqing, Sichuan, China | 25 | 1 | 7 | 27 | 1 | 6 | 106 | 2 | 207 |
| [209](http://pubmlst.org/perl/private/bigsdb/bigscurate.pl?page=info&db=pubmlst_csinensis_isolates&id=209) | CSSC7 | Cat, Chongqing, Sichuan, China | 71 | 1 | 4 | 5 | 1 | 17 | 29 | 2 | 208 |
| [210](http://pubmlst.org/perl/private/bigsdb/bigscurate.pl?page=info&db=pubmlst_csinensis_isolates&id=210) | CSSC8 | Cat, Chongqing, Sichuan, China | 72 | 9 | 1 | 8 | 1 | 90 | 14 | 1 | 209 |
| [211](http://pubmlst.org/perl/private/bigsdb/bigscurate.pl?page=info&db=pubmlst_csinensis_isolates&id=211) | CSSC9 | Cat, Chongqing, Sichuan, China | 73 | 2 | 1 | 10 | 27 | 33 | 10 | 11 | 210 |
| [212](http://pubmlst.org/perl/private/bigsdb/bigscurate.pl?page=info&db=pubmlst_csinensis_isolates&id=212) | CSSD1 | Cat, Taian, Shandong, China | 74 | 4 | 1 | 15 | 1 | 91 | 107 | 1 | 211 |
| [213](http://pubmlst.org/perl/private/bigsdb/bigscurate.pl?page=info&db=pubmlst_csinensis_isolates&id=213) | CSSD10 | Cat, Taian, Shandong, China | 65 | 13 | 11 | 28 | 3 | 63 | 71 | 1 | 212 |
| [214](http://pubmlst.org/perl/private/bigsdb/bigscurate.pl?page=info&db=pubmlst_csinensis_isolates&id=214) | CSSD15 | Cat, Taian, Shandong, China | 65 | 13 | 11 | 28 | 3 | 63 | 71 | 1 | 212 |
| [215](http://pubmlst.org/perl/private/bigsdb/bigscurate.pl?page=info&db=pubmlst_csinensis_isolates&id=215) | CSSD11 | Cat, Taian, Shandong, China | 44 | 13 | 11 | 16 | 1 | 63 | 71 | 1 | 213 |
| [216](http://pubmlst.org/perl/private/bigsdb/bigscurate.pl?page=info&db=pubmlst_csinensis_isolates&id=216) | CSSD12 | Cat, Taian, Shandong, China | 58 | 4 | 16 | 5 | 3 | 15 | 19 | 6 | 214 |
| [217](http://pubmlst.org/perl/private/bigsdb/bigscurate.pl?page=info&db=pubmlst_csinensis_isolates&id=217) | CSSD13 | Cat, Taian, Shandong, China | 44 | 13 | 11 | 3 | 1 | 63 | 71 | 1 | 215 |
| [218](http://pubmlst.org/perl/private/bigsdb/bigscurate.pl?page=info&db=pubmlst_csinensis_isolates&id=218) | CSSD14 | Cat, Taian, Shandong, China | 26 | 4 | 1 | 15 | 1 | 92 | 107 | 2 | 216 |
| [219](http://pubmlst.org/perl/private/bigsdb/bigscurate.pl?page=info&db=pubmlst_csinensis_isolates&id=219) | CSSD16 | Cat, Taian, Shandong, China | 23 | 1 | 6 | 3 | 3 | 6 | 107 | 1 | 217 |
| [220](http://pubmlst.org/perl/private/bigsdb/bigscurate.pl?page=info&db=pubmlst_csinensis_isolates&id=220) | CSSD17 | Cat, Taian, Shandong, China | 27 | 1 | 1 | 6 | 3 | 4 | 107 | 1 | 218 |
| [221](http://pubmlst.org/perl/private/bigsdb/bigscurate.pl?page=info&db=pubmlst_csinensis_isolates&id=221) | CSSD18 | Cat, Taian, Shandong, China | 64 | 8 | 1 | 3 | 1 | 93 | 108 | 4 | 219 |
| [222](http://pubmlst.org/perl/private/bigsdb/bigscurate.pl?page=info&db=pubmlst_csinensis_isolates&id=222) | CSSD19 | Cat, Taian, Shandong, China | 6 | 1 | 1 | 3 | 1 | 10 | 107 | 6 | 220 |
| [223](http://pubmlst.org/perl/private/bigsdb/bigscurate.pl?page=info&db=pubmlst_csinensis_isolates&id=223) | CSSD2 | Cat, Taian, Shandong, China | 2 | 1 | 1 | 5 | 3 | 94 | 108 | 6 | 221 |
| [224](http://pubmlst.org/perl/private/bigsdb/bigscurate.pl?page=info&db=pubmlst_csinensis_isolates&id=224) | CSSD3 | Cat, Taian, Shandong, China | 44 | 1 | 1 | 1 | 1 | 69 | 107 | 16 | 222 |
| [225](http://pubmlst.org/perl/private/bigsdb/bigscurate.pl?page=info&db=pubmlst_csinensis_isolates&id=225) | CSSD4 | Cat, Taian, Shandong, China | 31 | 1 | 1 | 29 | 1 | 6 | 107 | 6 | 223 |
| [226](http://pubmlst.org/perl/private/bigsdb/bigscurate.pl?page=info&db=pubmlst_csinensis_isolates&id=226) | CSSD6 | Cat, Taian, Shandong, China | 22 | 1 | 1 | 2 | 1 | 13 | 109 | 4 | 224 |
| [227](http://pubmlst.org/perl/private/bigsdb/bigscurate.pl?page=info&db=pubmlst_csinensis_isolates&id=227) | CSSD7 | Cat, Taian, Shandong, China | 5 | 1 | 1 | 3 | 1 | 13 | 109 | 1 | 225 |
| [228](http://pubmlst.org/perl/private/bigsdb/bigscurate.pl?page=info&db=pubmlst_csinensis_isolates&id=228) | CSSD8 | Cat, Taian, Shandong, China | 2 | 1 | 1 | 12 | 1 | 91 | 107 | 6 | 226 |
| [229](http://pubmlst.org/perl/private/bigsdb/bigscurate.pl?page=info&db=pubmlst_csinensis_isolates&id=229) | CSSD9 | Cat, Taian, Shandong, China | 31 | 1 | 3 | 8 | 1 | 6 | 110 | 1 | 227 |
| [230](http://pubmlst.org/perl/private/bigsdb/bigscurate.pl?page=info&db=pubmlst_csinensis_isolates&id=230) | CSSX10 | Cat, Linfen, Shanxi, China | 23 | 1 | 1 | 5 | 3 | 95 | 46 | 17 | 228 |
| [231](http://pubmlst.org/perl/private/bigsdb/bigscurate.pl?page=info&db=pubmlst_csinensis_isolates&id=231) | CSSX11 | Cat, Xianyang, Shannxi, China | 3 | 4 | 1 | 5 | 1 | 15 | 18 | 1 | 229 |
| [232](http://pubmlst.org/perl/private/bigsdb/bigscurate.pl?page=info&db=pubmlst_csinensis_isolates&id=232) | CSSX12 | Cat, Xianyang, Shannxi, China | 75 | 4 | 1 | 3 | 28 | 15 | 28 | 1 | 230 |
| [233](http://pubmlst.org/perl/private/bigsdb/bigscurate.pl?page=info&db=pubmlst_csinensis_isolates&id=233) | CSSX13 | Cat, Xianyang, Shannxi, China | 25 | 6 | 1 | 3 | 1 | 96 | 61 | 1 | 231 |
| [234](http://pubmlst.org/perl/private/bigsdb/bigscurate.pl?page=info&db=pubmlst_csinensis_isolates&id=234) | CSSX14 | Cat, Xianyang, Shannxi, China | 2 | 1 | 1 | 6 | 3 | 4 | 111 | 1 | 232 |
| [235](http://pubmlst.org/perl/private/bigsdb/bigscurate.pl?page=info&db=pubmlst_csinensis_isolates&id=235) | CSSX15 | Cat, Xianyang, Shannxi, China | 55 | 1 | 1 | 5 | 1 | 4 | 44 | 1 | 233 |
| [236](http://pubmlst.org/perl/private/bigsdb/bigscurate.pl?page=info&db=pubmlst_csinensis_isolates&id=236) | CSSX16 | Cat, Xianyang, Shannxi, China | 32 | 1 | 1 | 3 | 1 | 4 | 112 | 1 | 234 |
| [237](http://pubmlst.org/perl/private/bigsdb/bigscurate.pl?page=info&db=pubmlst_csinensis_isolates&id=237) | CSSX17 | Cat, Xianyang, Shannxi, China | 1 | 6 | 1 | 8 | 1 | 96 | 113 | 9 | 235 |
| [238](http://pubmlst.org/perl/private/bigsdb/bigscurate.pl?page=info&db=pubmlst_csinensis_isolates&id=238) | CSSX19 | Cat, Xianyang, Shannxi, China | 31 | 1 | 1 | 8 | 1 | 4 | 111 | 1 | 236 |
| [239](http://pubmlst.org/perl/private/bigsdb/bigscurate.pl?page=info&db=pubmlst_csinensis_isolates&id=239) | CSSX20 | Cat, Xianyang, Shannxi, China | 50 | 1 | 1 | 7 | 1 | 4 | 114 | 4 | 237 |
| [240](http://pubmlst.org/perl/private/bigsdb/bigscurate.pl?page=info&db=pubmlst_csinensis_isolates&id=240) | CSSX4 | Cat, Linfen, Shanxi, China | 2 | 1 | 1 | 29 | 1 | 4 | 114 | 18 | 238 |
| [241](http://pubmlst.org/perl/private/bigsdb/bigscurate.pl?page=info&db=pubmlst_csinensis_isolates&id=241) | CSSX5 | Cat, Linfen, Shanxi, China | 1 | 4 | 1 | 5 | 29 | 15 | 115 | 4 | 239 |
| [242](http://pubmlst.org/perl/private/bigsdb/bigscurate.pl?page=info&db=pubmlst_csinensis_isolates&id=242) | CSSX6 | Cat, Linfen, Shanxi, China | 27 | 1 | 1 | 29 | 3 | 4 | 116 | 2 | 240 |
| [243](http://pubmlst.org/perl/private/bigsdb/bigscurate.pl?page=info&db=pubmlst_csinensis_isolates&id=243) | CSSX7 | Cat, Linfen, Shanxi, China | 76 | 1 | 10 | 8 | 29 | 6 | 27 | 4 | 241 |
| [244](http://pubmlst.org/perl/private/bigsdb/bigscurate.pl?page=info&db=pubmlst_csinensis_isolates&id=244) | CSSX8 | Cat, Linfen, Shanxi, China | 25 | 1 | 1 | 3 | 1 | 4 | 111 | 19 | 242 |
| [245](http://pubmlst.org/perl/private/bigsdb/bigscurate.pl?page=info&db=pubmlst_csinensis_isolates&id=245) | CSSX9 | Cat, Linfen, Shanxi, China | 6 | 1 | 1 | 5 | 30 | 4 | 44 | 19 | 243 |
| [246](http://pubmlst.org/perl/private/bigsdb/bigscurate.pl?page=info&db=pubmlst_csinensis_isolates&id=246) | CSZJ1 | Cat, Shanghai, Zhejiang, China | 8 | 4 | 1 | 9 | 31 | 97 | 95 | 1 | 244 |
| [247](http://pubmlst.org/perl/private/bigsdb/bigscurate.pl?page=info&db=pubmlst_csinensis_isolates&id=247) | CSZJ10 | Cat, Shanghai, Zhejiang, China | 27 | 1 | 1 | 5 | 31 | 4 | 117 | 1 | 245 |
| [248](http://pubmlst.org/perl/private/bigsdb/bigscurate.pl?page=info&db=pubmlst_csinensis_isolates&id=248) | CSZJ11 | Cat, Shanghai, Zhejiang, China | 47 | 1 | 1 | 3 | 31 | 10 | 57 | 6 | 246 |
| [249](http://pubmlst.org/perl/private/bigsdb/bigscurate.pl?page=info&db=pubmlst_csinensis_isolates&id=249) | CSZJ12 | Cat, Shanghai, Zhejiang, China | 2 | 1 | 1 | 5 | 3 | 13 | 118 | 1 | 247 |
| [250](http://pubmlst.org/perl/private/bigsdb/bigscurate.pl?page=info&db=pubmlst_csinensis_isolates&id=250) | CSZJ13 | Cat, Shanghai, Zhejiang, China | 6 | 1 | 1 | 5 | 2 | 98 | 23 | 1 | 248 |
| [251](http://pubmlst.org/perl/private/bigsdb/bigscurate.pl?page=info&db=pubmlst_csinensis_isolates&id=251) | CSZJ15 | Cat, Shanghai, Zhejiang, China | 8 | 1 | 1 | 3 | 1 | 99 | 15 | 6 | 249 |
| [252](http://pubmlst.org/perl/private/bigsdb/bigscurate.pl?page=info&db=pubmlst_csinensis_isolates&id=252) | CSZJ16 | Cat, Shanghai, Zhejiang, China | 15 | 1 | 1 | 5 | 2 | 100 | 119 | 1 | 250 |
| [253](http://pubmlst.org/perl/private/bigsdb/bigscurate.pl?page=info&db=pubmlst_csinensis_isolates&id=253) | CSZJ2 | Cat, Shanghai, Zhejiang, China | 3 | 1 | 1 | 1 | 5 | 34 | 1 | 1 | 251 |
| [254](http://pubmlst.org/perl/private/bigsdb/bigscurate.pl?page=info&db=pubmlst_csinensis_isolates&id=254) | CSZJ3 | Cat, Shanghai, Zhejiang, China | 25 | 1 | 1 | 5 | 6 | 33 | 14 | 1 | 252 |
| [255](http://pubmlst.org/perl/private/bigsdb/bigscurate.pl?page=info&db=pubmlst_csinensis_isolates&id=255) | CSZJ4 | Cat, Shanghai, Zhejiang, China | 58 | 2 | 1 | 3 | 1 | 101 | 81 | 1 | 253 |
| [256](http://pubmlst.org/perl/private/bigsdb/bigscurate.pl?page=info&db=pubmlst_csinensis_isolates&id=256) | CSZJ8 | Cat, Shanghai, Zhejiang, China | 1 | 1 | 1 | 5 | 3 | 100 | 53 | 6 | 254 |
