# Supplementary material for: Low Divergence of Clonorchis sinensis in China Based on Multilocus Analysis
Source: PLoS One. 2013 Jun 18;8(6):e67006. doi: 10.1371/journal.pone.0067006 (PMC3688995; doi:10.1371/journal.pone.0067006)
Supplement: Table S2 — Primers used in this study. (DOC) [file pone.0067006.s004.doc]

Table S2. Primers used in this study.

| **Gene** | **Primer** | **Oligo nucleotides sequences (5’-3’)** | **Reference** |
| --- | --- | --- | --- |
| ITS1 | ITS1R | CGATTCTAGTTCCGTCATCT | Liu W et al. Acta Tropica, 2007, 101:91-94 |
|  | ITS1F | CCGCTCAGAGTTGTACTCAT |  |
| Actin | ActinR | ATGGGTGATGAGGACGTTGCAGCT | This study |
|  | ActinF | CATGATCGAGTTGTACGTCGTCTC |  |
| Pm-Int | PME-FW | GCAGAGAATATGCGACTCAAG | Sergei V et al. Parasitol Res. 2009.106:293–297 |
|  | PMI-RV | AAATTATCCCGTTCCGCTTC |  |
| Cox1 | JB3 | TTTTTTGGGCATCCTGAGGTTTAT | Bowles J et al. Molecular phylogenetics and evolution. 1995,4:103-109 |
|  | JB4.5 | TAAAGAAAGAACATAATGAAAATG |  |
| EF-1a | CsEF-R | ATTGTCAGTGAGGATCCCAGT | This study |
|  | CsEF-F | TCCAGCCGGGTCGCACAATCT |  |
| Tubulin | CsTub-F | GCTTACTCATTCCCTTGGTG | This study |
|  | CsTub-R | ATCTCGTCCATACCTTCTCC |  |
| Cox3 | CsCox3-F | TTTTATTCCTGGGTGAG | This study |
|  | CsCox3-R | TGCTTTGATTTATACGC |  |
| NADH3 | CsNADH3-F | GTTTTGTTGTATTTGCAG | This study |
|  | CsNADH3-R | ATAACCTTGACTCACCTC |  |
| NADH4 | CsNADH4-F | CCCTGCTTTGTTACTGCT | This study |
|  | CsNADH4-R | ATATCTCAATGCCGCCTC |  |
| NADH5 | CsNADH5-F | GTTGCGGTAAGGAGGATC | This study |
|  | CsNADH5-R | GGTAAGCGACACGAGAAG |  |
| NADH6 | CsNADH6-F | TTTCGTGGTATCTGTTGGTG | This study |
|  | CsNADH6-R | AAAGAGTCCTTCTCCCTCCC |  |
| GAPDH | CsGAPDH-F | ACTGGGATCCGAGATGTCCAAACCTAAG | This study |
|  | CsGAPDH-R | GCGCCTCGAGCCATTCTTCTTGAATTTA |  |
